# Supplementary figures and images for: Paracrine regulation of neural crest EMT by placodal MMP28
Source: PLoS Biol. 2023 Aug 17;21(8):e3002261. doi: 10.1371/journal.pbio.3002261 (PMC10479893; doi:10.1371/journal.pbio.3002261)

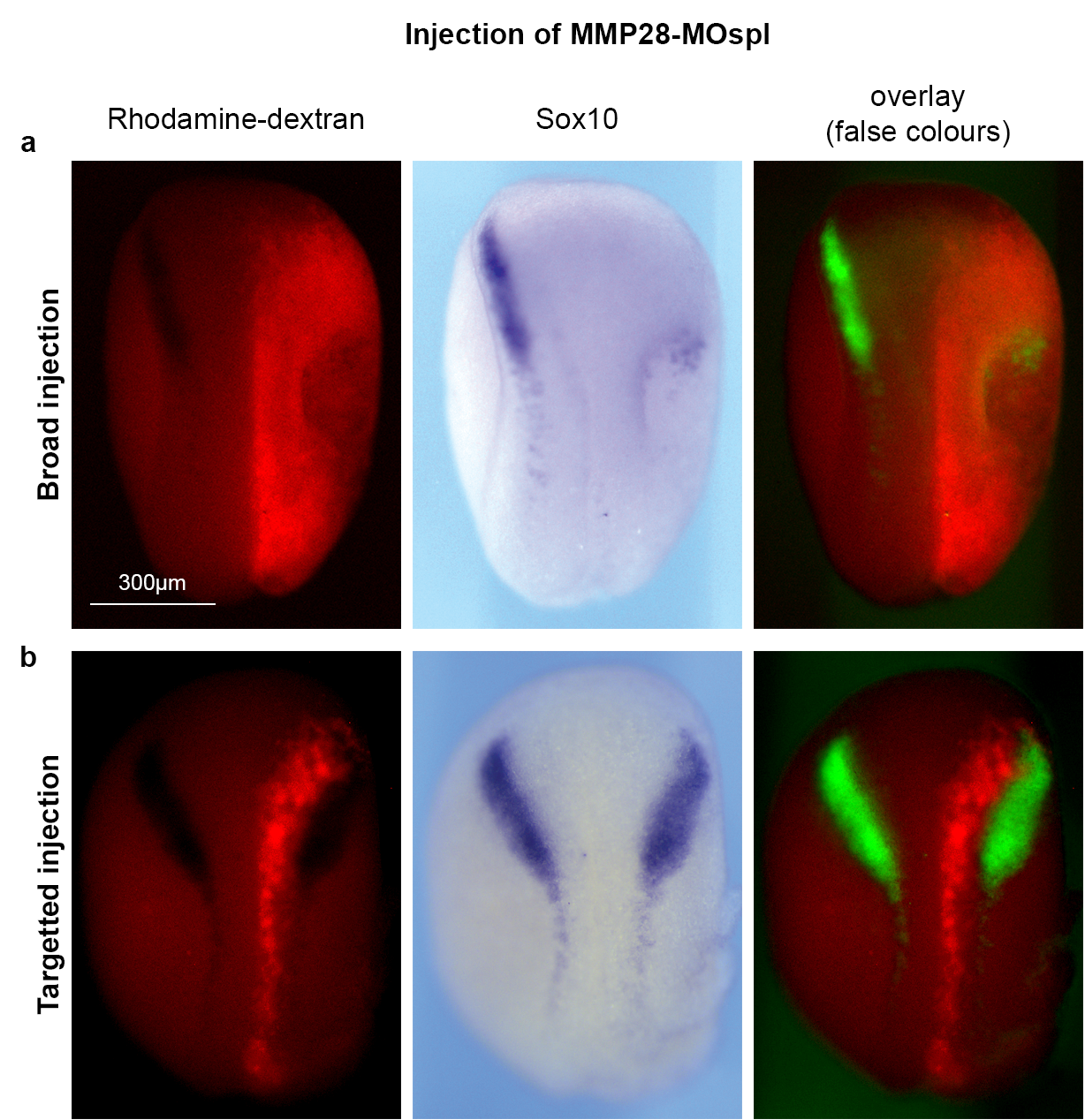

Supplement: S1 Fig — (a, b) Representative images of embryos injected with MMP28-MOspl in a broad manner (a, n = 15), overlapping with the placodal domain or in a targeted manner (b, n = 15) targeting the neural fold and medial crest subregion but excluding the placodes, analysed by in situ hybridization against Sox10. MO was co-injected with rhodamine-dextran for tracing (red). (TIF) [file pbio.3002261.s001.tif]

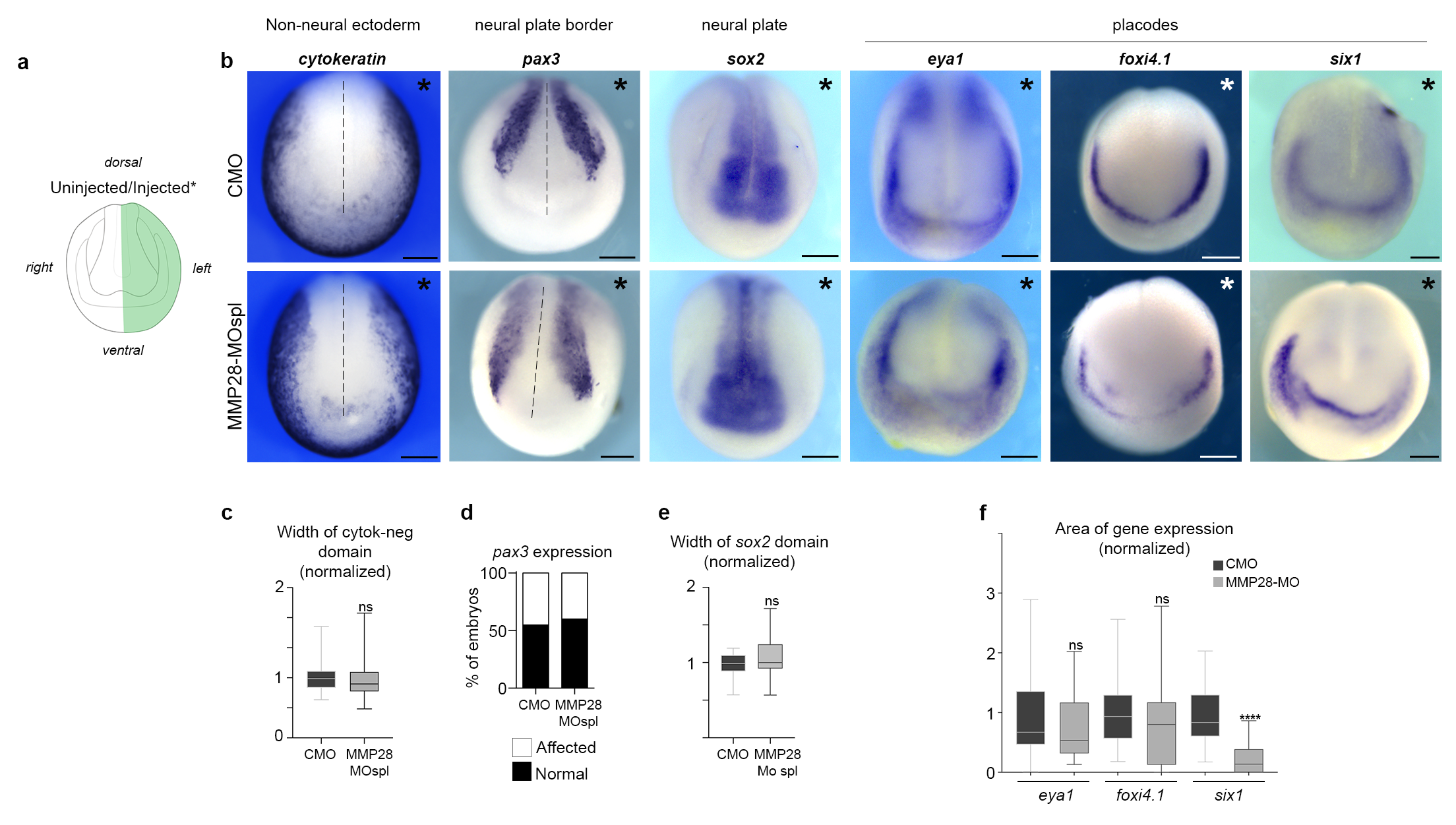

Supplement: S2 Fig — (a) Diagram representing the anterior view of Xenopus laevis neurula (Stage 16). Embryos were injected on their left-hand side (green). (b) Representative images of embryos injected with either control MO (CMO) or MMP28-MOspl after in situ hybridization for cytokeratin, pax3, sox2, eya1, foxi4.1 and six1, asterisks mark the injected side. (c) Ratio of the width of the cytokeratin-negative domain from the midline for the injected and uninjected sides, normalised to the CMO condition; CMO (n = 43), MMP28MOspl (n = 63). (d) Percentages of embryos with normal or affected expression of pax3 after injection of CMO (n = 40) or MMP28-MOspl (n = 20). (e) Ratio of the width of the sox2-positive domain from the midline for the injected and uninjected sides, normalised to the CMO condition; (CMO n = 20), MMP28MOspl (n = 15). (f) Ratio of area of gene expression for the injected and uninjected sides, normalised to the CMO condition; eya1: (CMO n = 15), MMP28MOspl (n = 14); foxi4.1: CMO (n = 27), MMP28MOspl (n = 37); six1: CMO (n = 20), MMP28MOspl (n = 49). Statistics: Student t tests c, p = 0.3510; e, p = 0.7125; f, eya1, p = 0.3572; foxi4.1, p = 0.3512; six1, p < 0.0001. For panel (d), contingency table T = 0.135, α = ns. Scale bar, 200 μm. (TIF) [file pbio.3002261.s002.tif]

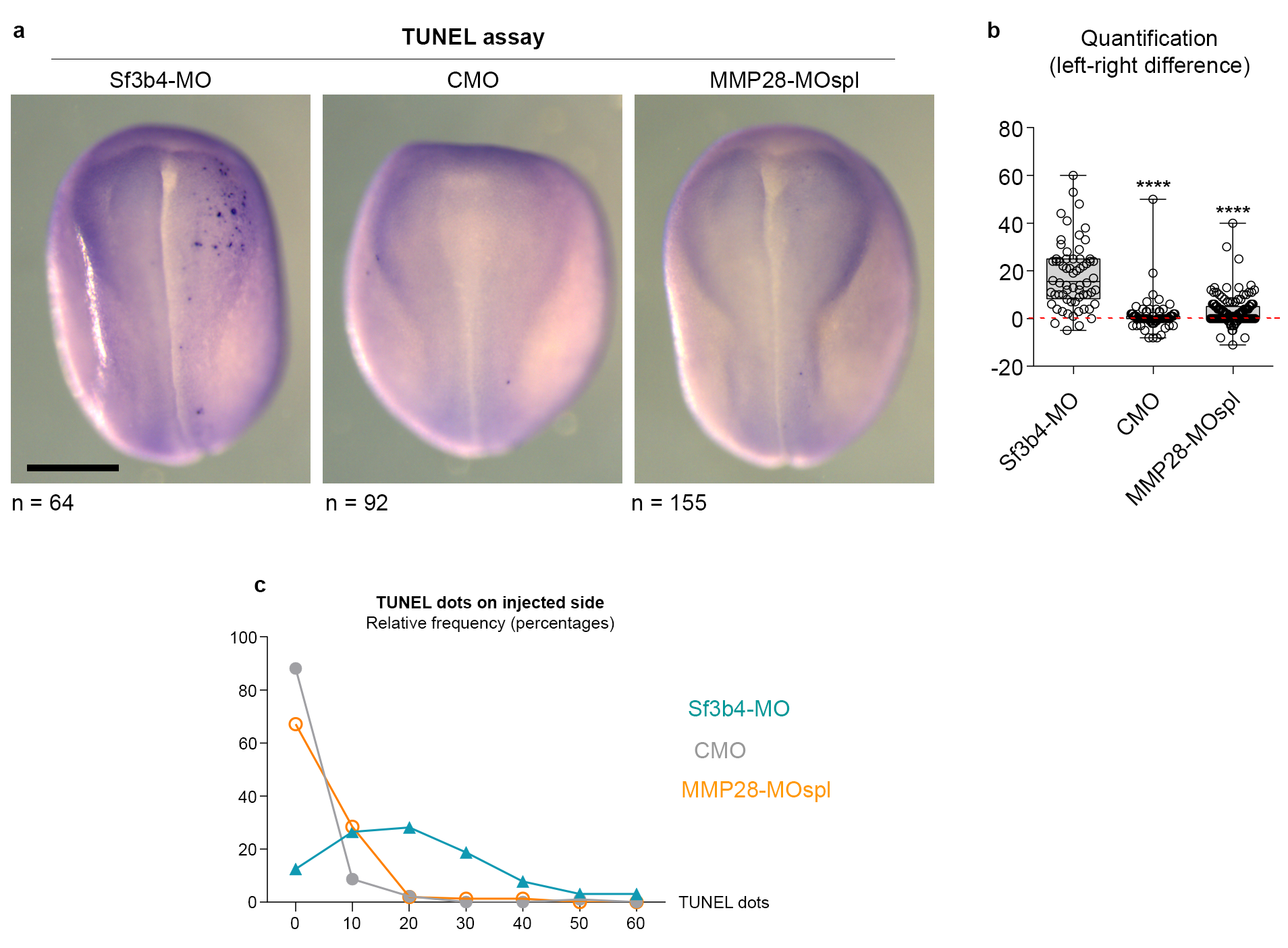

Supplement: S3 Fig — (a) Representative images of a TUNEL assay in embryos injected with 10 ng of Morpholino against Sf3b4 (used as a positive control for induction of cell death, see Materials and methods), 4 ng of control MO or 4 ng of MMP28spl-MO in 2 blastomeres at 8-cell stage. (b) Differences between the number of TUNEL dots on each side within the neural crest region. The neural crest region was defined as the lateral half of each anterior neural fold. A value of 0 means that each side had the same number of TUNEL dots on either side. A negative value indicates more cell death on the non-injected side than the injected side while a positive value indicates more cell death on the injected side. (c) Frequency distribution of TUNEL dots in the neural crest region on the injected side in all conditions. Respectively 96% and 94% of all embryos injected with CMO or MMP28spl-MO only had between 0 and 20 TUNEL dots in the injected neural crest region, the remaining 4/6% had more than 20 TUNEL dots. By contrast, after inhibition of Sf3b4, only 37% of embryos had between 0 and 20 TUNEL dots in injected neural crest cells, 47% had more than 20 dots, and the remaining 8% had 30 dots or more on the injected side. ANOVA, Kruskal–Wallis; **** p < 0.0001. (TIF) [file pbio.3002261.s003.tif]

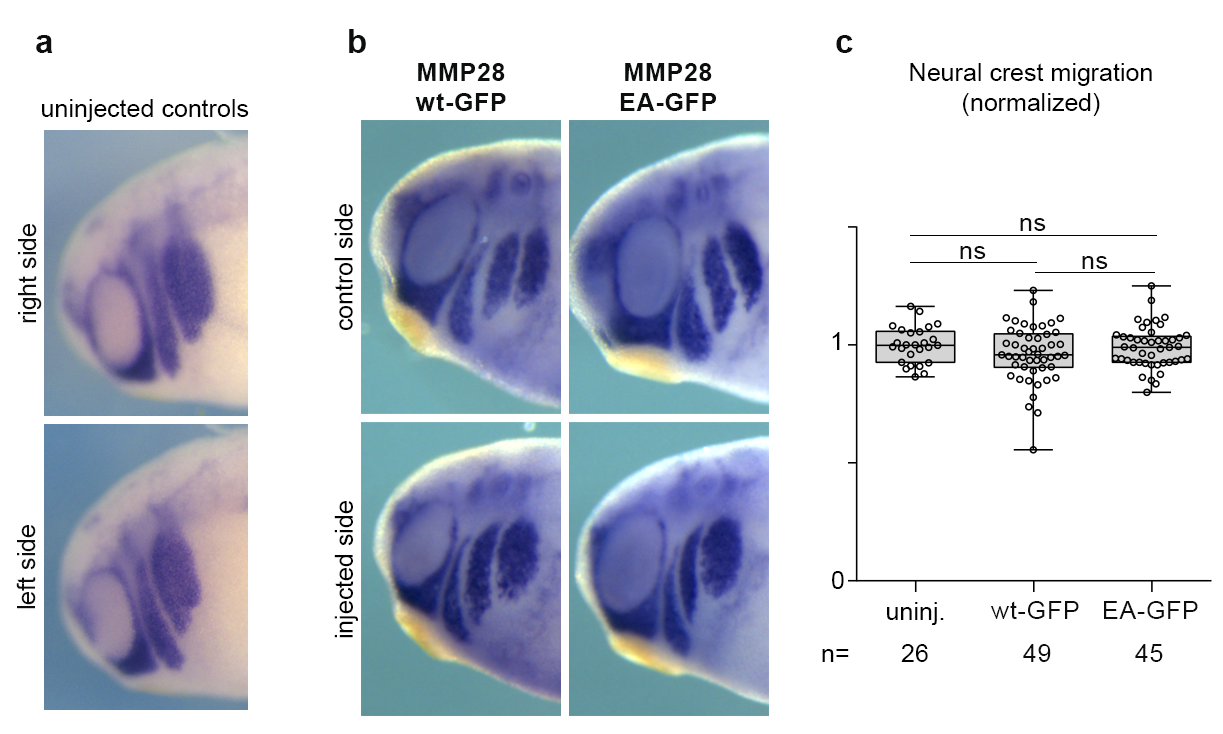

Supplement: S4 Fig — (a, b) In situ hybridization for Twist in uninjected embryos (a) and embryos injected with 900 pg MMP28wt-GFP or MMP28-EA-GFP mRNA (b). (c) Mean dorsoventral migration of neural crest cells in uninjected controls or after MMP28 overexpression, normalised to control side. ANOVA, followed by multiple comparisons. Uninjected vs. MMP28wt-GFP, p = 0.132 (ns); uninjected vs. MMP28-EA-GFP, p = 0.699 (ns); MMP28wt-GFP vs. MMP28-EA-GFP, p = 0.189 (ns). Note that MMP28 overexpression does not affect neural crest migration and does not induce ectopic Twist expression. (TIF) [file pbio.3002261.s004.tif]

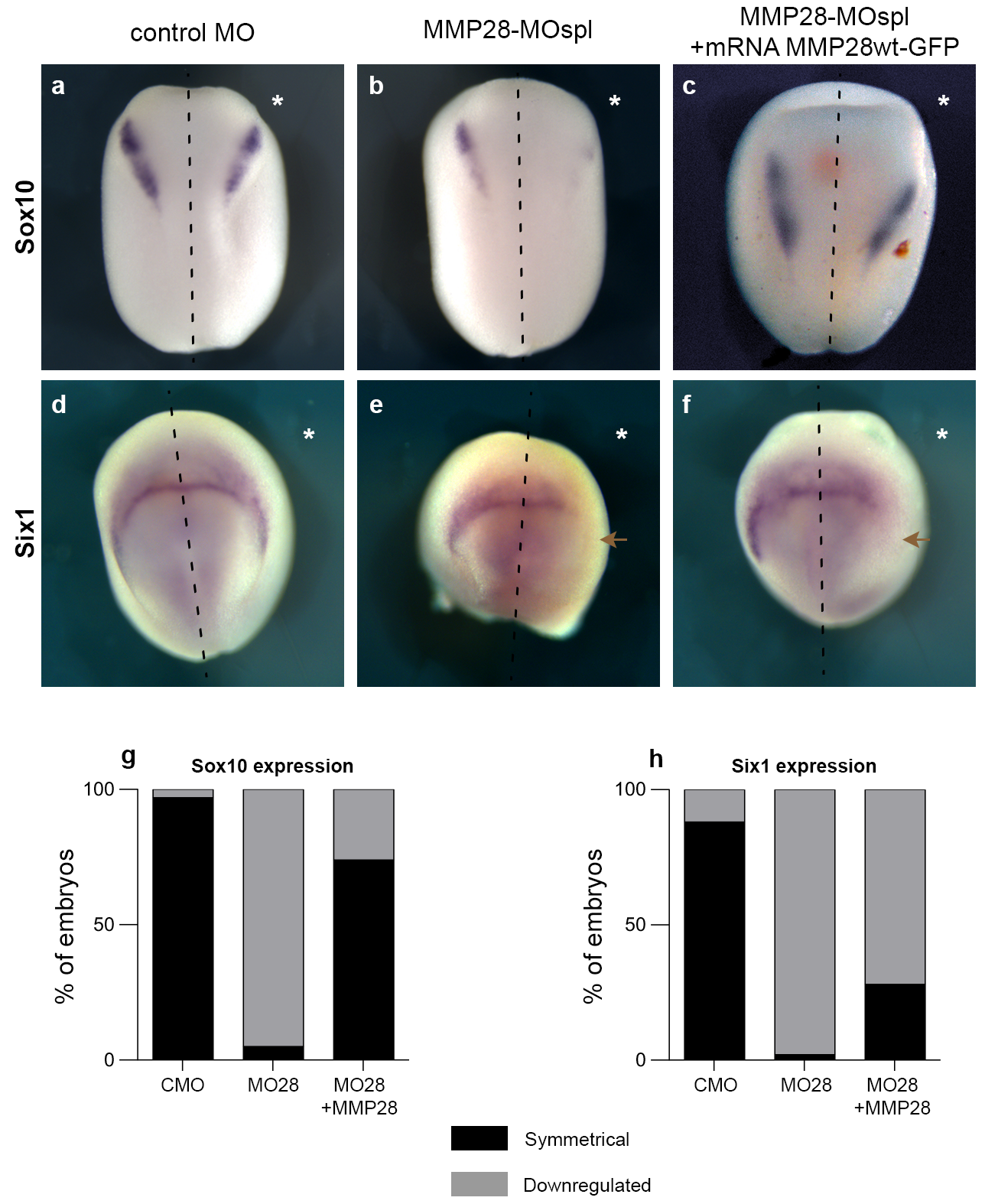

Supplement: S5 Fig — (a–f) In situ hybridization for Sox10 (a–c) and Six1 (d–f), in embryos injected with CMO (a, n = 29; d, n = 56), MMP28-MOspl (b, n = 40; e, n = 43), or co-injected with MMP28-MOspl and mRNA for MMP28wt-GFP (c, n = 31; f, n = 41). (g, h) Proportions of embryos with symmetrical or decreased expression of Sox10 (g) or Six1 (h) in each experimental condition. Contingency tables for comparison of proportions: Sox10 CMO vs. MOspl, T = 57.34 (***), MOspl vs. rescue condition, T = 36.65 (***); Six1 CMO vs. MOspl, T = 70.59 (***), MOspl vs. rescue condition, T = 7.7 (***), Rescue Sox10 vs. Rescue Six1, T = 19.5 (***). Asterisks on images indicate the injected side. Dotted lines mark the midline of each embryo. Brown arrows indicate the missing portion of Six1 expression domain in MMP28-MOspl and MMP28-MOspl+mRNA MMP28wt-GFP embryos. Scale, embryos are 500 μm wide on average. (TIF) [file pbio.3002261.s005.tif]

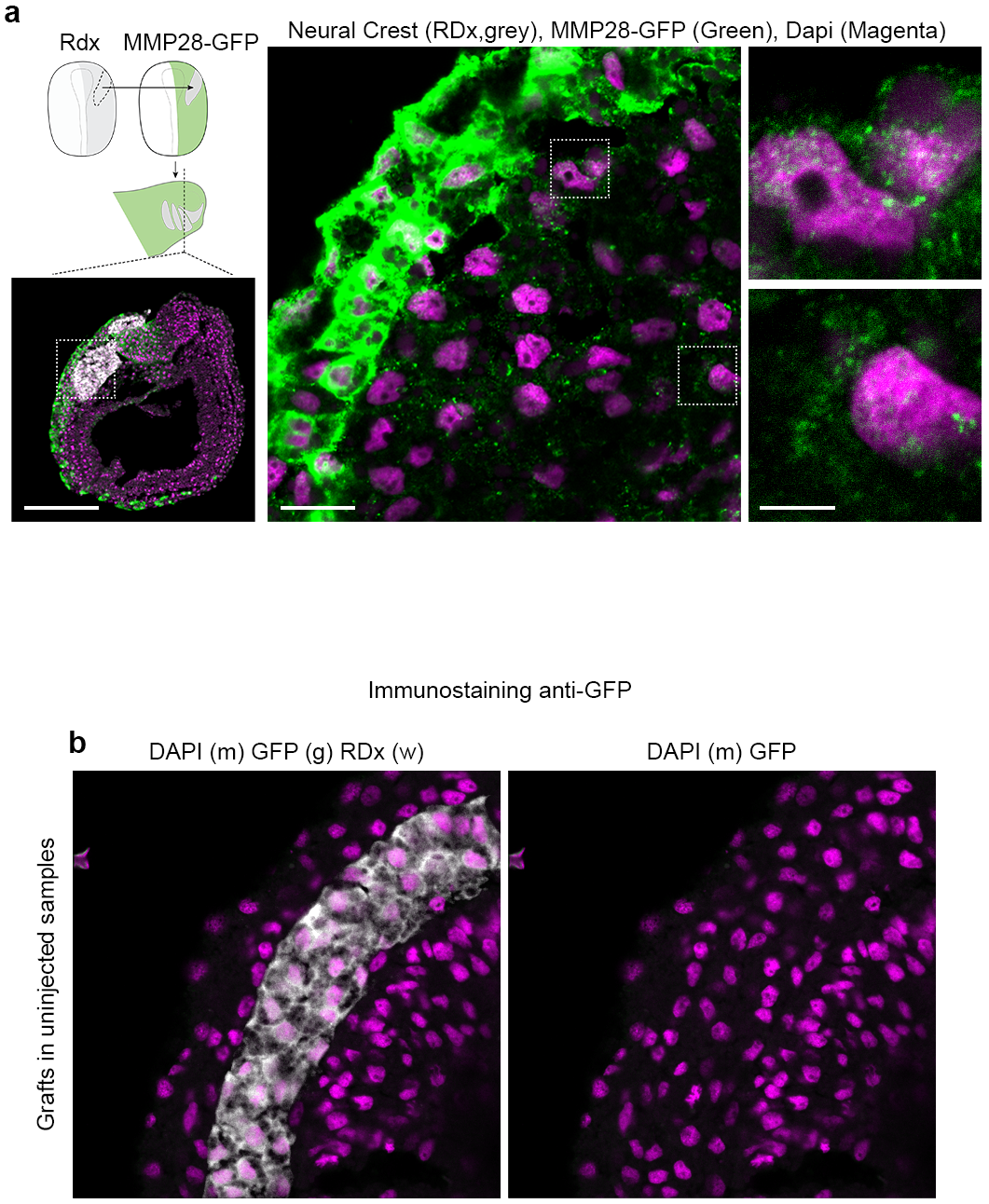

Supplement: S6 Fig — (a) Immunostaining against-GFP on cryosections of embryos expression MMP28wt-GFP in which rhodamine-dextran (RDx) positive control neural crest cells were grafted. (b) Immunostaining against-GFP on cryosections of non-injected embryos (negative controls) in which rhodamine-dextran positive neural crest cells were grafted. Nuclei were counterstained with DAPI (magenta); immunostaining for GFP is shown in green and rhodamine-dextran in grey. Scale bars, panel (a) 200 μm (low magnification), 20 μm (high magnification), and 5 μm on zooms. Note that in absence of GFP, the GFP immunostaining gives no significant signal. (TIF) [file pbio.3002261.s006.tif]

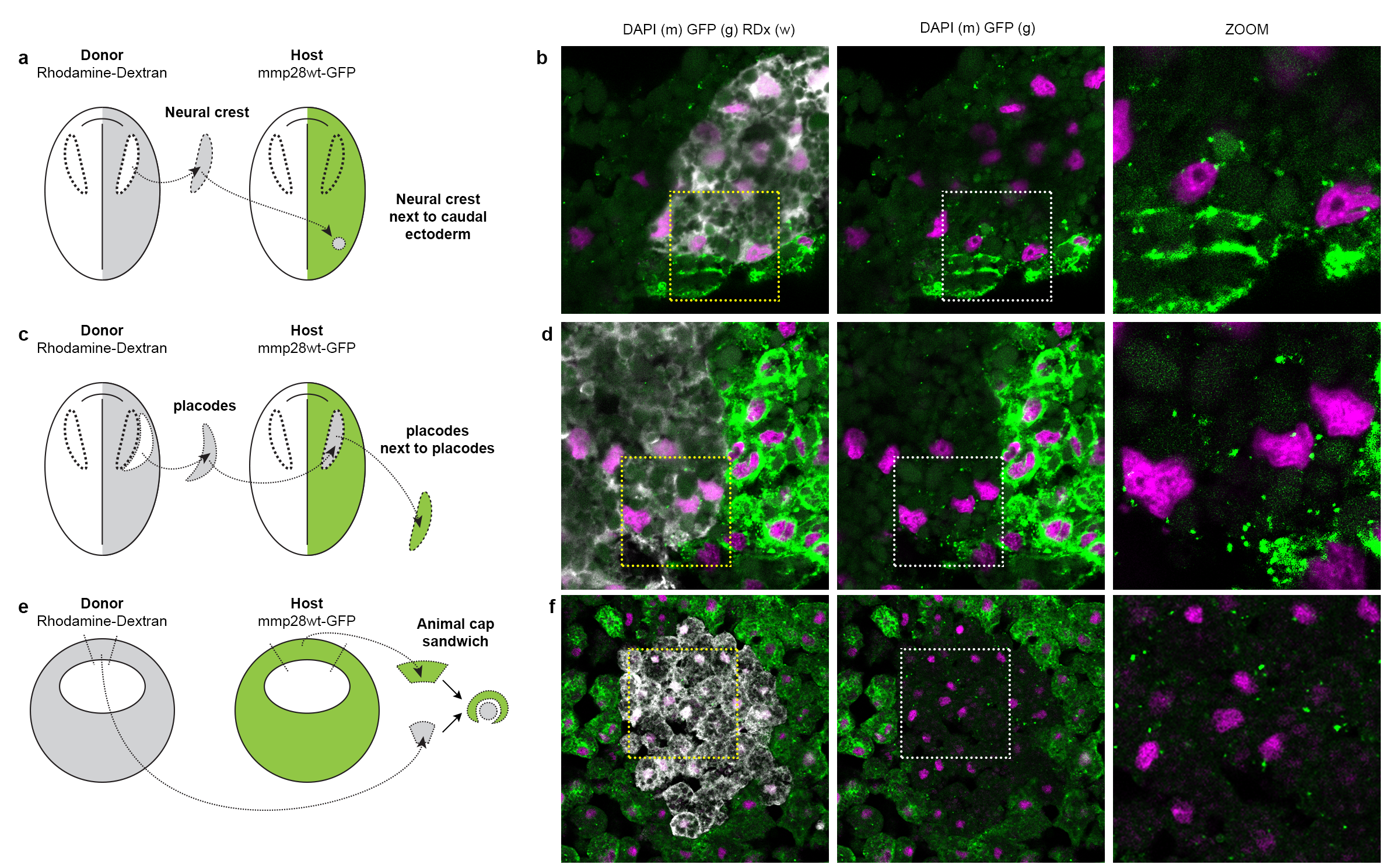

Supplement: S7 Fig — (a, b) Graft of neural crest cells labelled with rhodamine-dextran next to caudal ectoderm expressing MMP28-GFP. (c, d) Graft of placodals cells labelled with rhodamine-dextran next to placodal cells expressing MMP28-GFP. (e, f) Animal cap sandwiches between animal caps expressing MMP28-GFP and animal caps labelled with rhodamine-dextran. Counterstaining with DAPI (magenta). Rhodamine-dextran (grey), MMP28-GFP (green). No anti-GFP staining was performed on these samples. Scale is given by the nuclei that have a diameter of 12 μm on average. Numbers and quantifications of internalisation and nuclear import are shown on Fig 5, panel (c). (TIF) [file pbio.3002261.s007.tif]

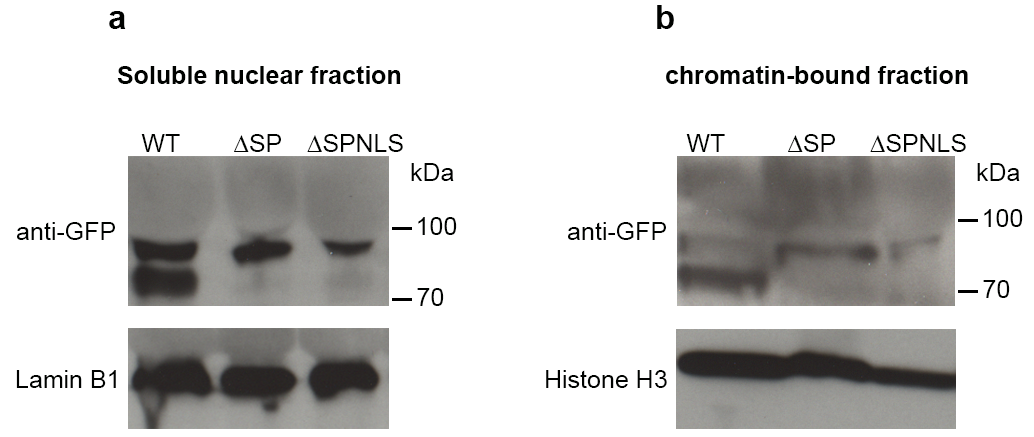

Supplement: S8 Fig — (a, b) Western blots using anti-GFP antibody after cell fractionation from embryos expressing MMP28-GFP (WT), MMP28-ΔSP (deletion of the secretion peptide), or MMP28-ΔSPNLS (deletion of the secretion peptide and insertion of a strong NLS in C-terminus) on the soluble (a) and chromatin-associated (b) nuclear fractions, representative image from 2 independent experiments. Lamin B1 and Histone H3 were used as controls for the soluble and chromatin-associated fractions, respectively. Note that the lower band of MMP28 (circa 70kDa) is not detected in the ΔSP and ΔSPNLS conditions indicating that the pro-domain of MMP28 is not removed if MMP28 is prevented from entering the secretion pathway. (TIF) [file pbio.3002261.s008.tif]

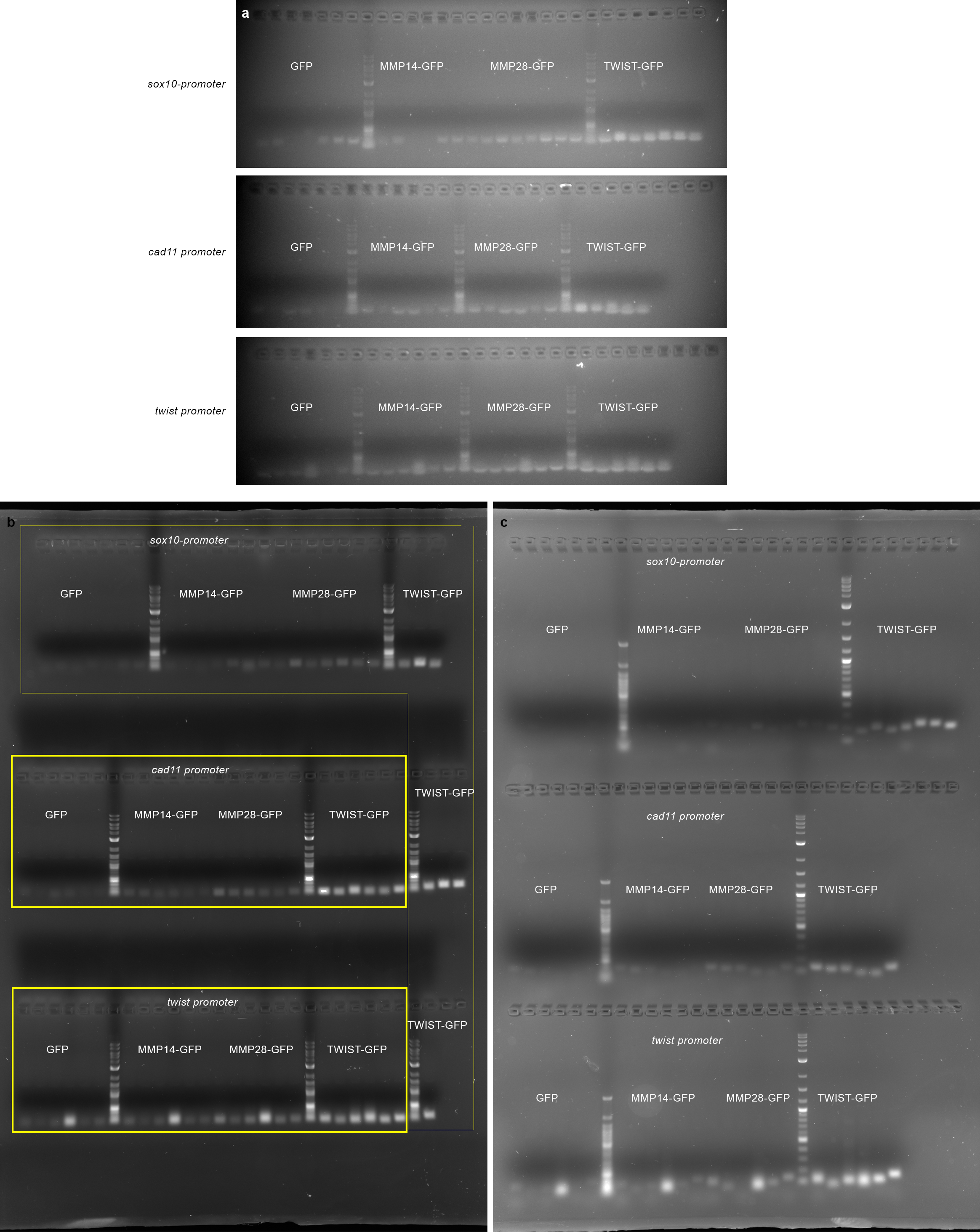

Supplement: S9 Fig — (TIF) [file pbio.3002261.s009.tif]

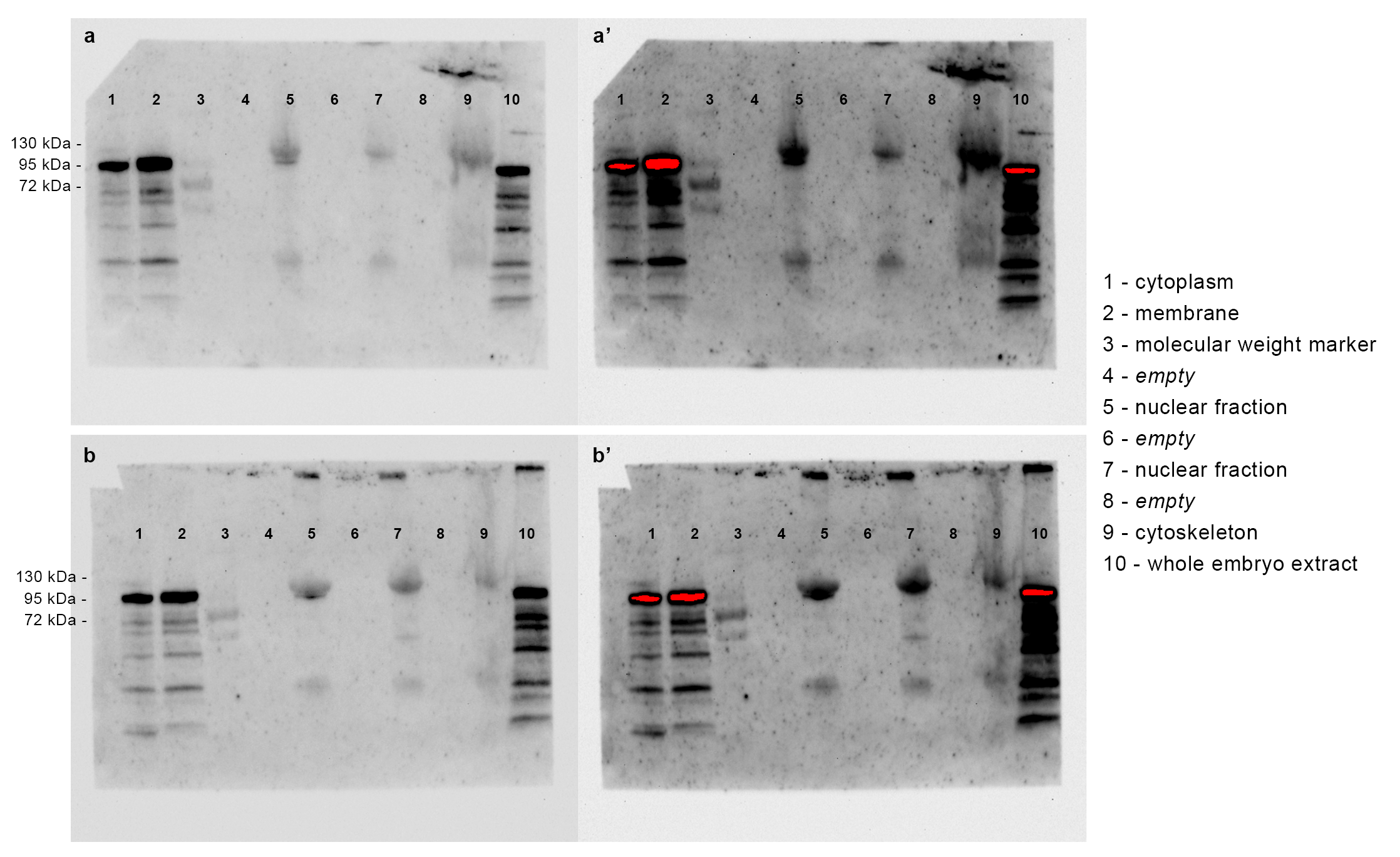

Supplement: S10 Fig — (a–b’) Western blots using anti-GFP antibody after cell fractionation from embryos expressing MMP14-GFP; (a) and (b) are 2 fractionations from independent samples, and (a’) and (b’) are the same blots as (a) and (b) with exposure time optimised for band detection in the nuclear fractions. (TIF) [file pbio.3002261.s010.tif]

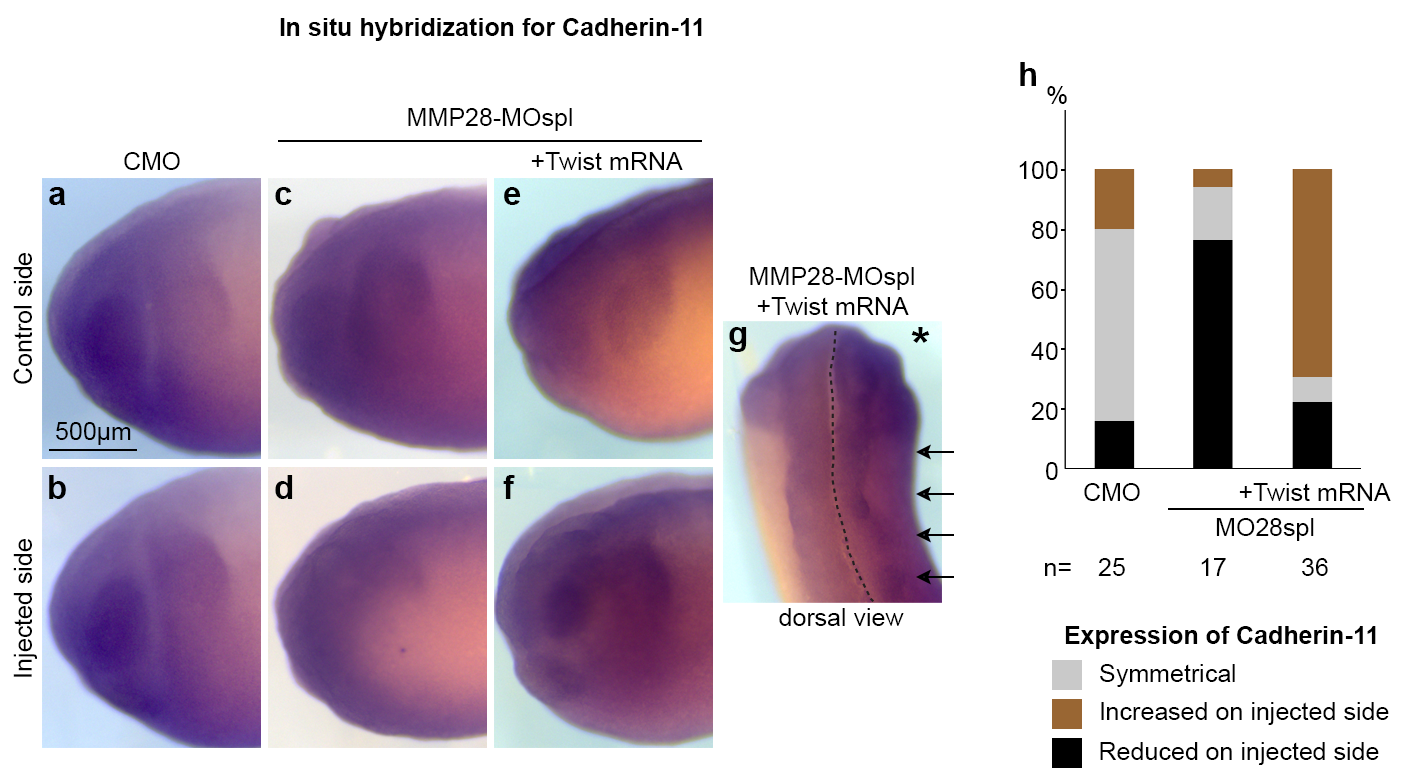

Supplement: S11 Fig — (a, b) Embryos injected with control MO. (c, d) Embryos injected with MMP-MOspl. (c–f) Embryos injected with MMP28-MOspl and Twist mRNA. (g) Dorsal view of the embryo shown in (e) and (f). (h) Percentage of embryos with expression of Cadherin-11 that is either symmetrical (grey) or increased/reduced (brown/black) on the injected side. Note that all embryos that had an increased expression of Cadherin-11 in the MMP28-MOspl+Twist mRNA condition also had ectopic expression of Cadherin-11 in the ectoderm as seen on panel (g) (arrows). Asterisk indicates the injected side. (TIF) [file pbio.3002261.s011.tif]

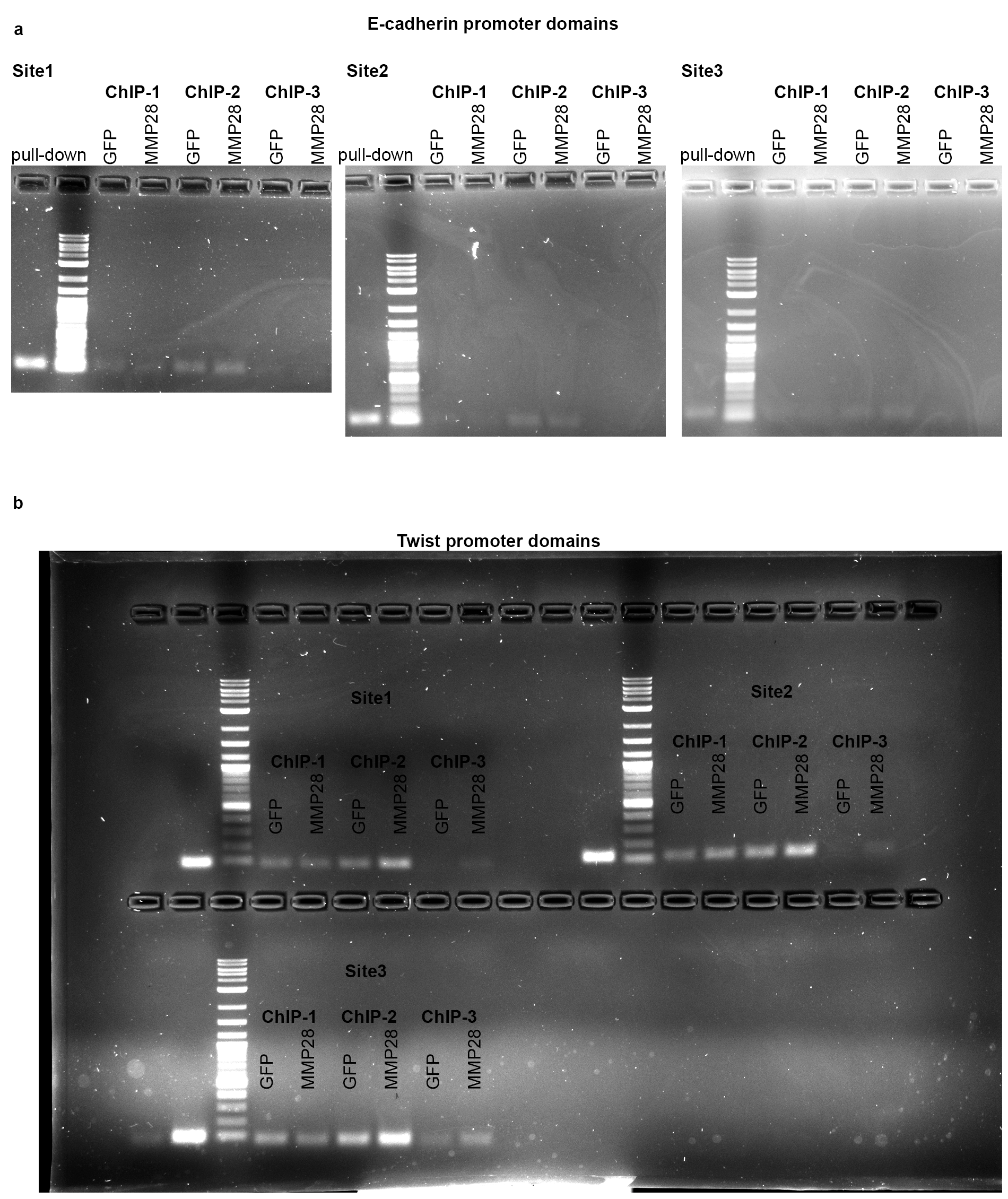

Supplement: S12 Fig — (a, b) Original images for the 3 independent ChIP assays analysed in Fig 7G–7L. On each gel, the band located left to the marker of size is the positive control of PCR efficiency for each site on total chromatin extracts. (TIF) [file pbio.3002261.s012.tif]
