# Supplementary material for: Paracrine regulation of neural crest EMT by placodal MMP28
Source: PLoS Biol. 2023 Aug 17;21(8):e3002261. doi: 10.1371/journal.pbio.3002261 (PMC10479893; doi:10.1371/journal.pbio.3002261)
Supplement: S1 Raw Images — (PDF) [file pbio.3002261.s019.pdf]

S1_raw_images

Paracrine regulation of neural crest EMT by placodal MMP28

Nadège Gouignard[1,2], Anne Bibonne[1], Joao Mata[3], Fernanda Bajanca[1], Bianka Berki[1], Elias Barriga[3], Jean-Pierre Saint-Jeannet[2] and Eric Theveneau[1]*

1, Centre de Biologie du Développement (CBD), Centre de Biologie Intégrative (CBI), Université de Toulouse, CNRS, UPS, 118 route de Narbonne 31062, Toulouse Cedex 09, France.
2, New York University, College of Dentistry, Department of Molecular Pathobiology, 345E 24th street, New York NY 10010, USA.
3, Instituto Gulbenkian de Ciência, Mechanisms of Morphogenesis Lab, Oeiras, Portugal.

Corresponding author: eric.theveneau@univ-tlse3.fr

FIGURE 1g

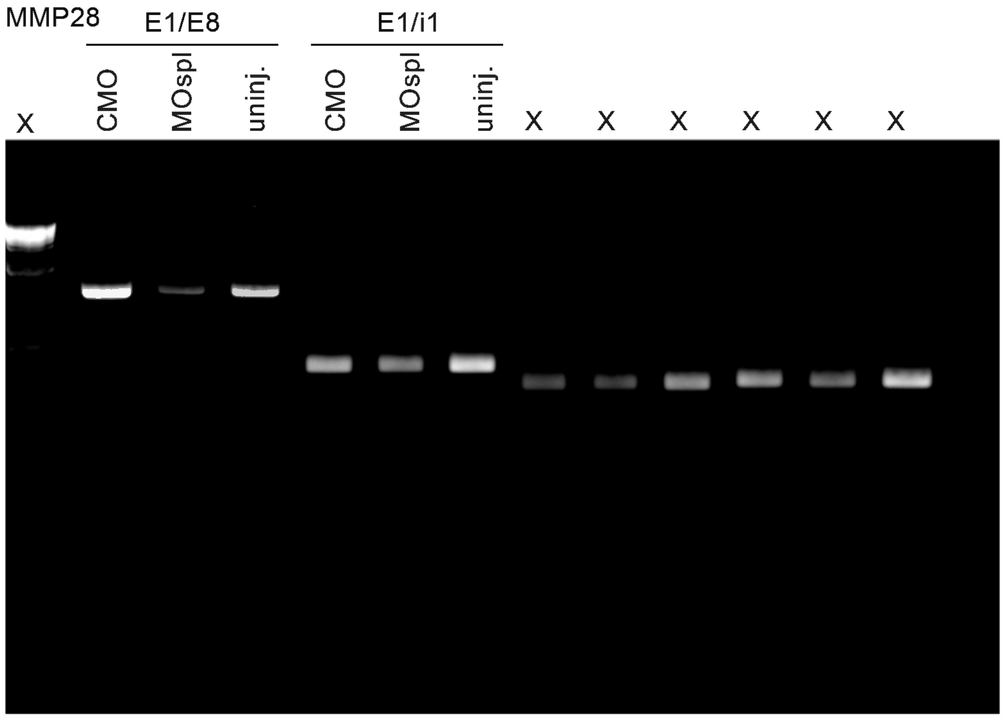

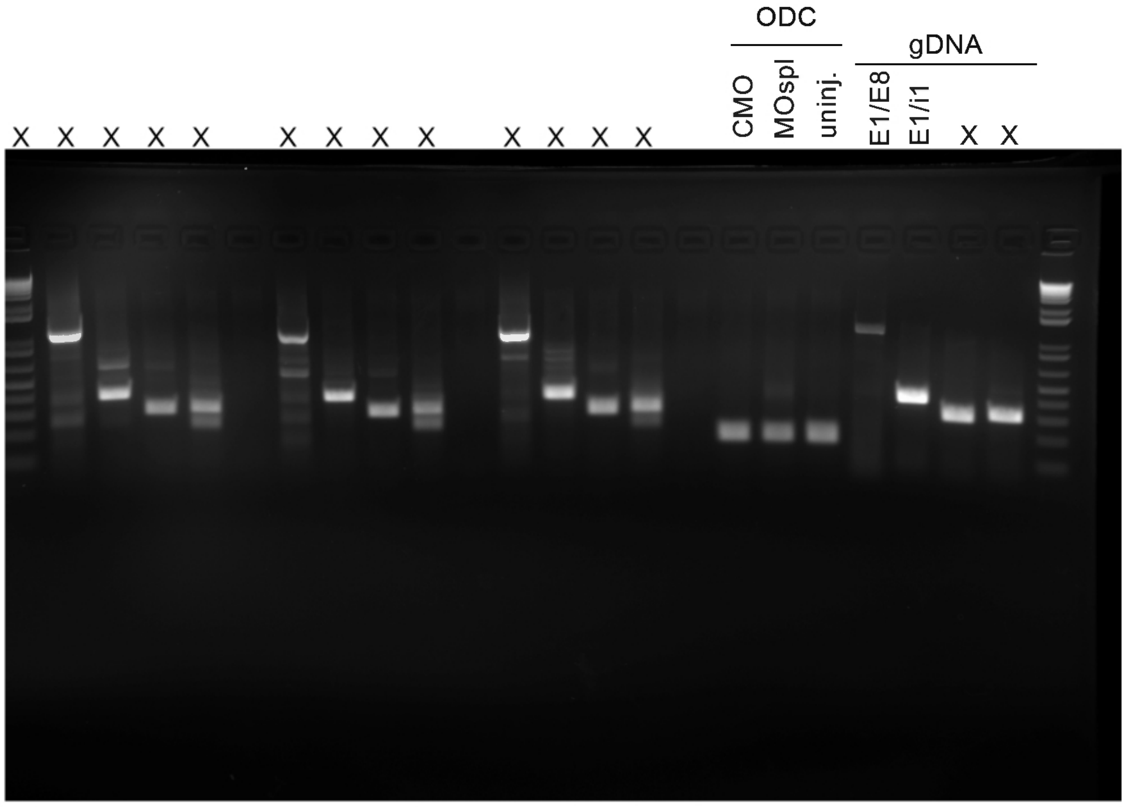

Images acquired on gel imager ChemiDoc XRS+ from Biorad

Note: to generate the blank gDNA E1/E8 panel on Figure 1g an empty part of the lane, close to the well, was cropped as the expected size of such fragment from genomic DNA is around 40 000 bp and cannot be amplified via regular PCR. Also note that a non-specific band appears at circa 1000bp.

FIGURE 4

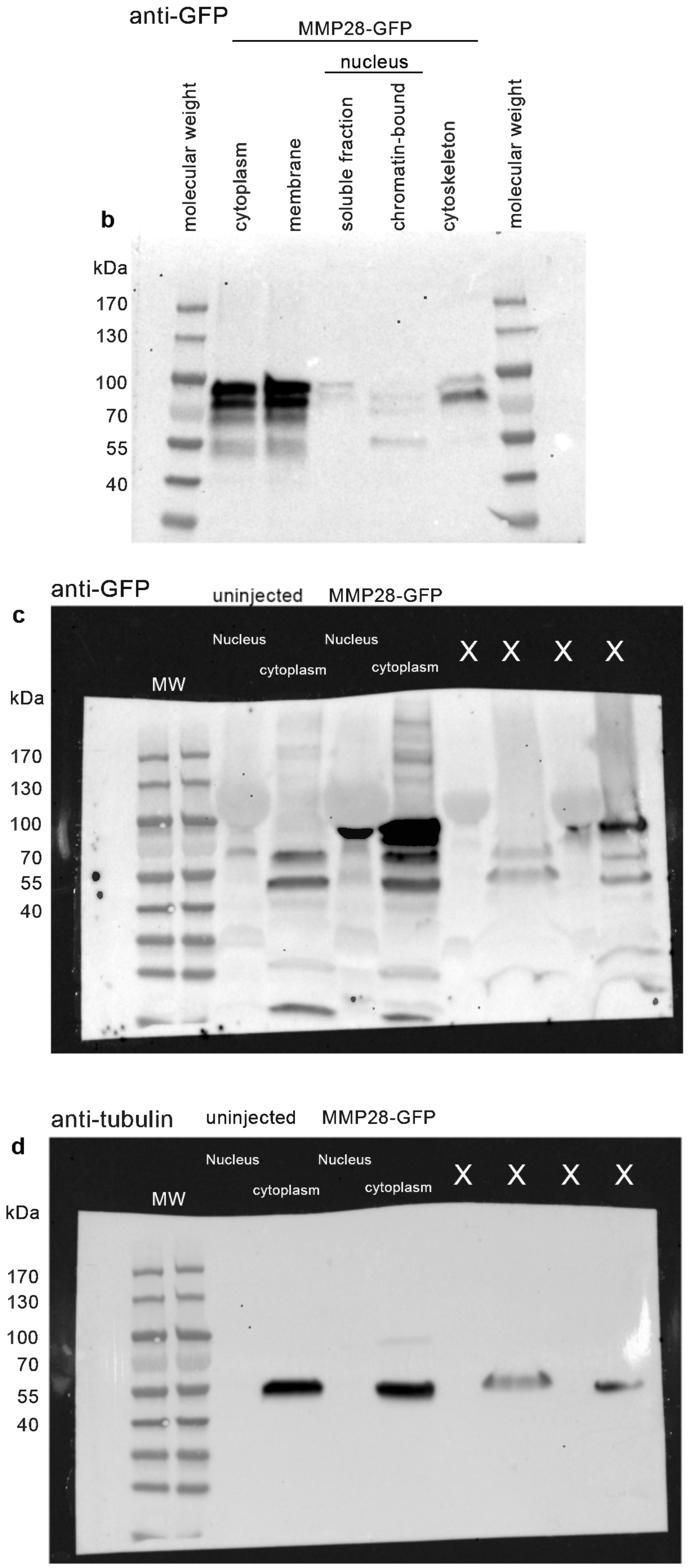

Images acquired on ChemiDoc XRS+ from Biorad.

Supporting Figure 8

panel a

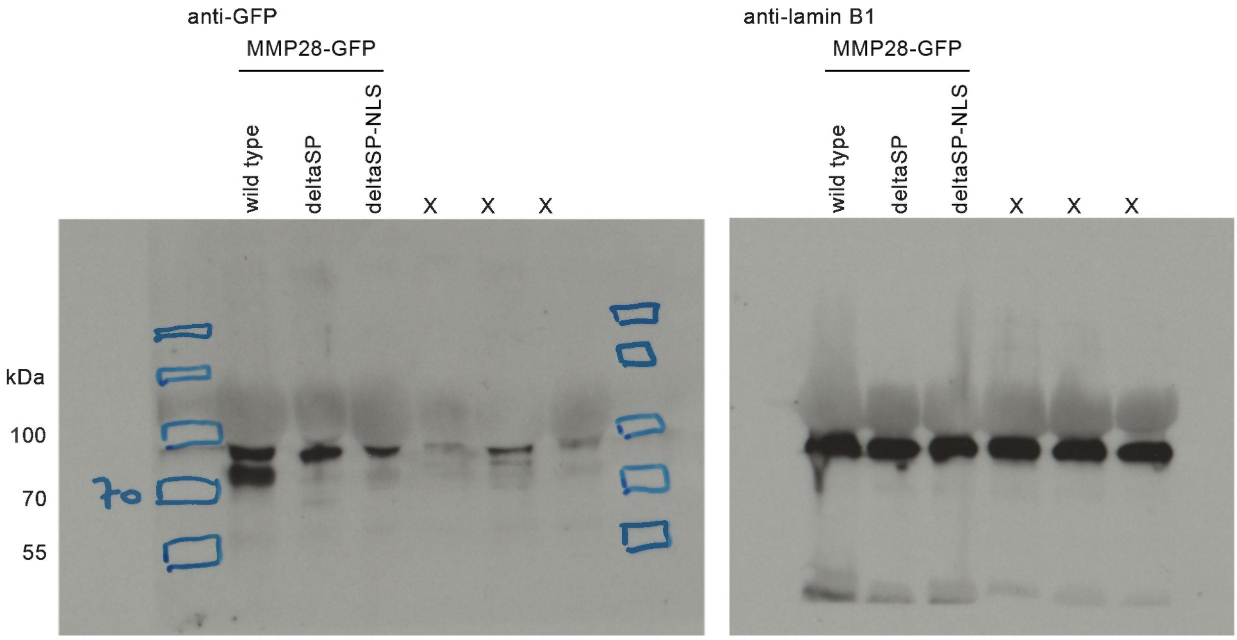

panel b

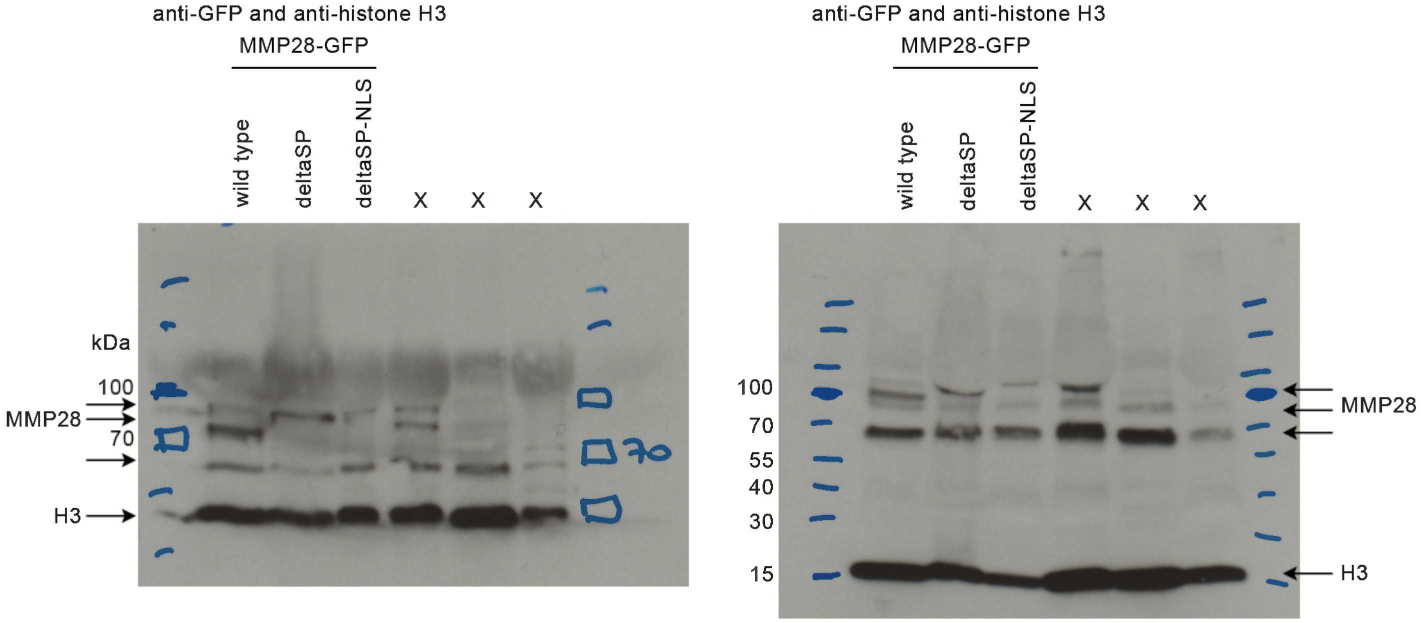

Blots were developped on film and digitalized using a high resolution scanner

Supporting Figure 9

panel a

For all conditions loading order of PCR product is from proximal to distal sites.
Only the 3 most proximal sites were used in the analysis

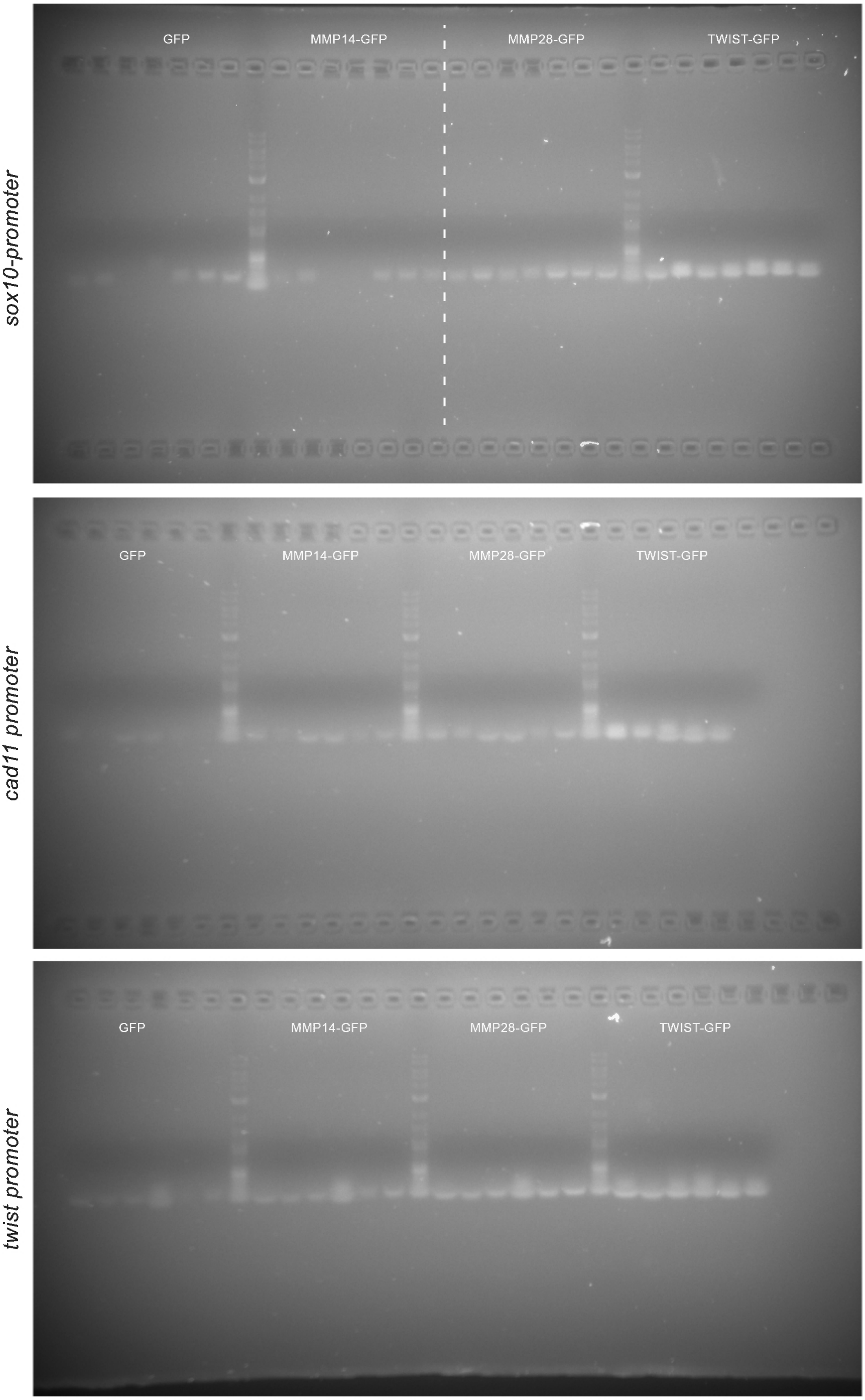

The gel is stained with XpertGreen according to manufacture protocol. Samples are mixed with 6x loading Orange-G.
Imaged in Auto mode in a BioRad GelDoc Imaging System

Supporting Figure 9 (continued)

panel b

For all conditions loading order of PCR product is from proximal to distal sites.
Only the 3 most proximal sites were used in the analysis

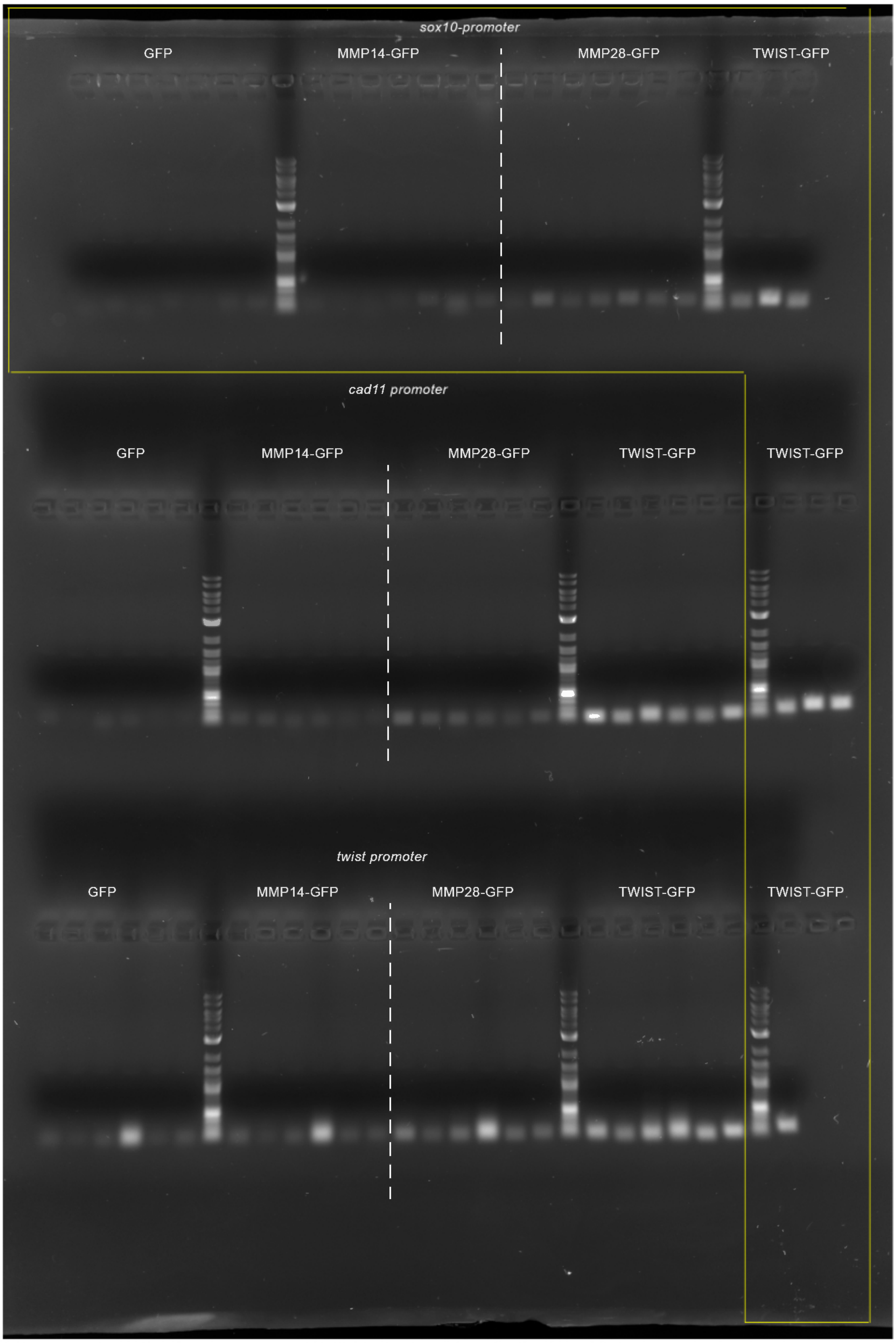

The gel is stained with XpertGreen according to manufacture protocol. Samples are mixed with 6x loading Orange-G.
Imaged in Auto mode in a BioRad GelDoc Imaging System

Supporting Figure 9 (continued)

panel c

For all conditions loading order of PCR product is from proximal to distal sites.
Only the 3 most proximal sites were used in the analysis

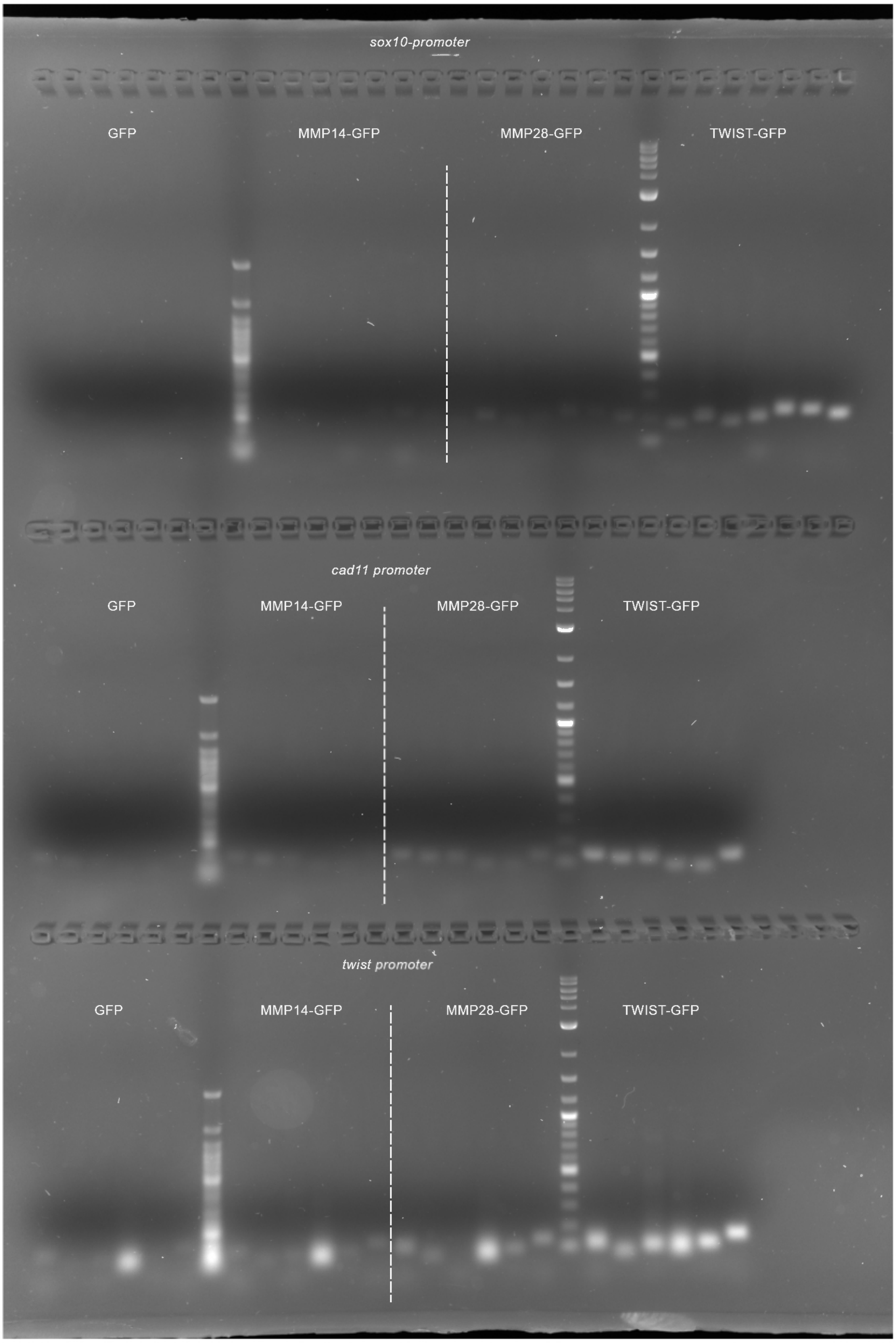

The gel is stained with XpertGreen according to manufacture protocol. Samples are mixed with 6x loading Orange-G. imaged in Auto mode in a BioRad GelDoc Imaging System

Supporting Figure 12

panel a

E-cadherin promoter domains

Site1

ChIP-1 **ChIP-2** **ChIP-3**

pull-down GFP MMP28 GFP MMP28 GFP MMP28 **x** **x**

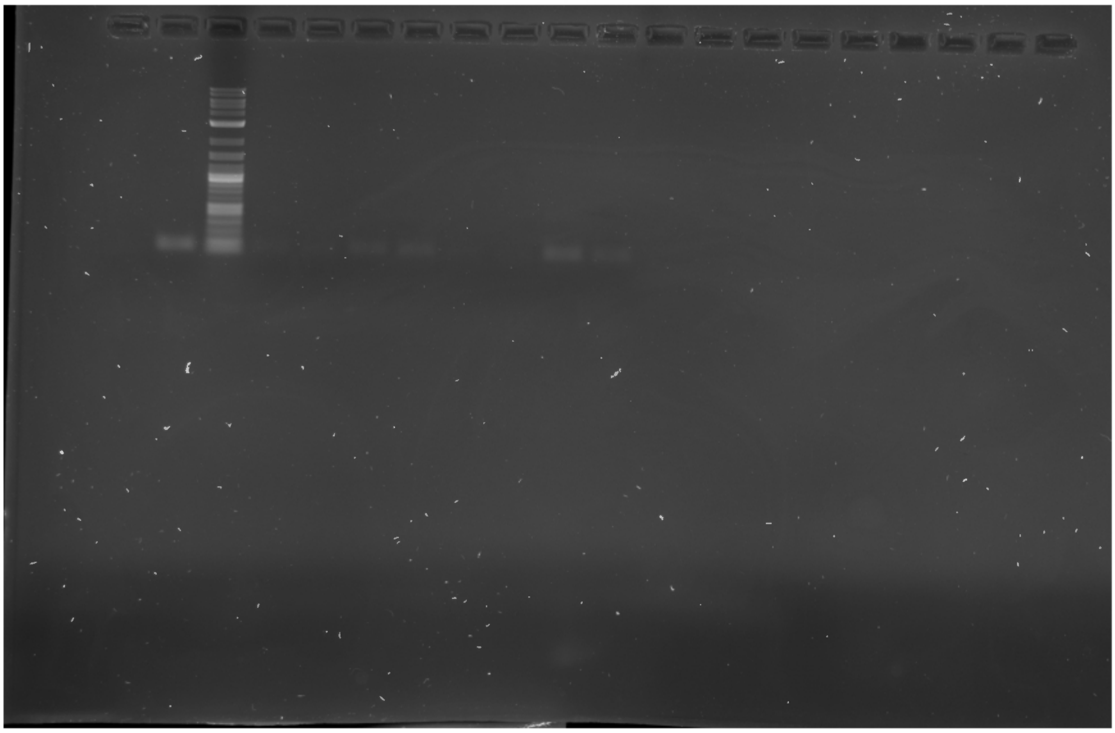

Site2

ChIP-1 **ChIP-2** **ChIP-3**

pull-down GFP MMP28 GFP MMP28 GFP MMP28 **x** **x**

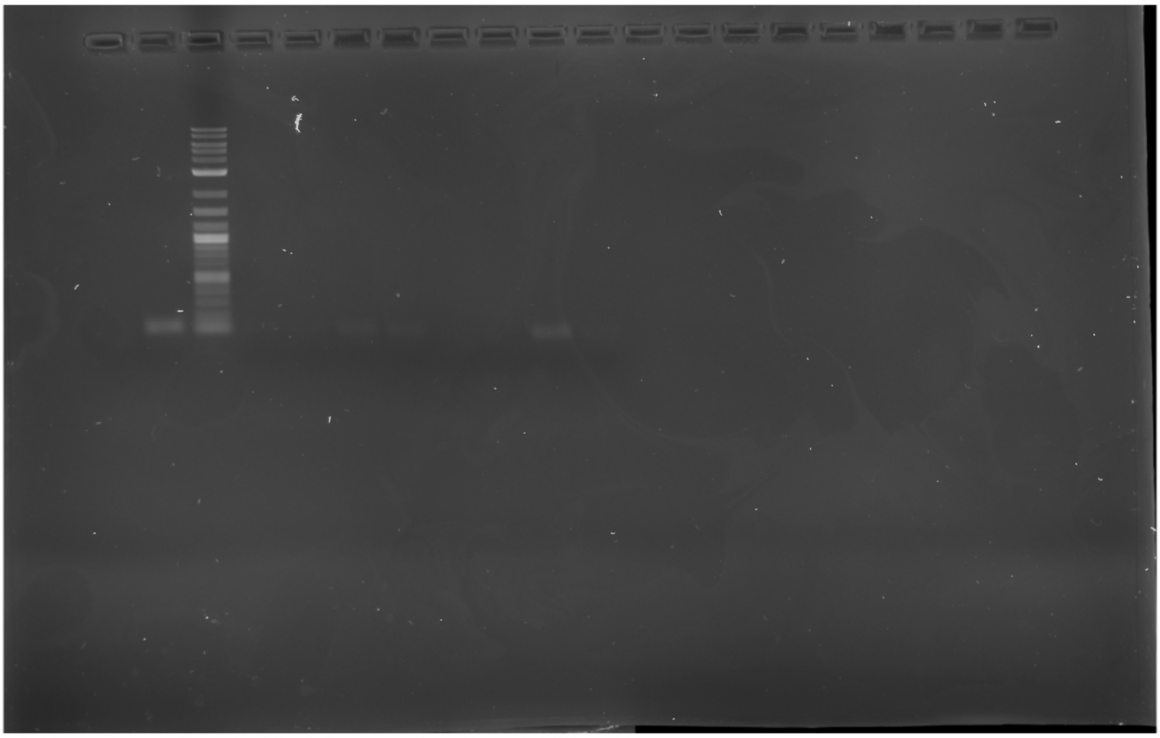

The gel is stained with XpertGreen according to manufacture protocol. Samples are mixed with 6x loading Orange-G. Imaged in Auto mode in a BioRad GelDoc Imaging System

Supporting Figure 12 (continued)

panel a

E-cadherin promoter domains

Site3

ChIP-1 **ChIP-2** **ChIP-3**

pull-down GFP MMP28 GFP MMP28 GFP MMP28 **X** **X**

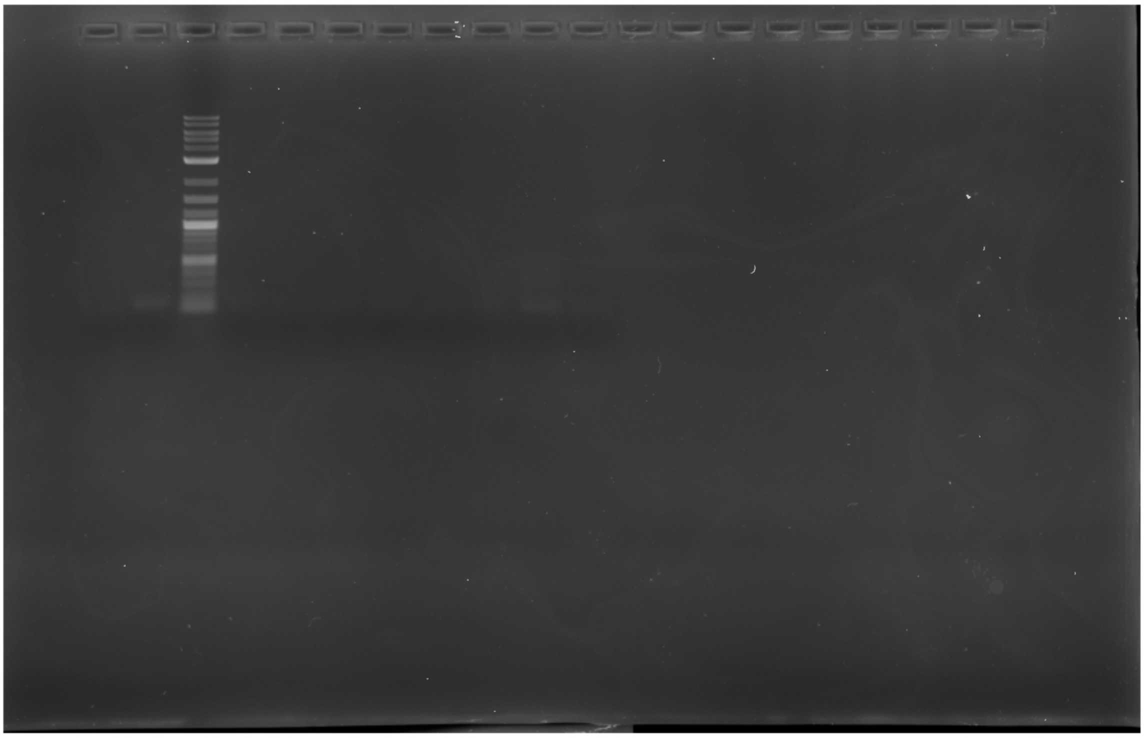

The gel is stained with XpertGreen according to manufacture protocol. Samples are mixed with 6x loading Orange-G. Imaged in Auto mode in a BioRad GelDoc Imaging System

Supporting Figure 12 (continued)

panel b

Twist promoter domains

Site1 | **Site2**

ChIP-1 **ChIP-2** **ChIP-3** | **ChIP-1** **ChIP-2** **ChIP-3**

GFP MMP28 GFP MMP28 GFP MMP28 | GFP MMP28 GFP MMP28 GFP MMP28

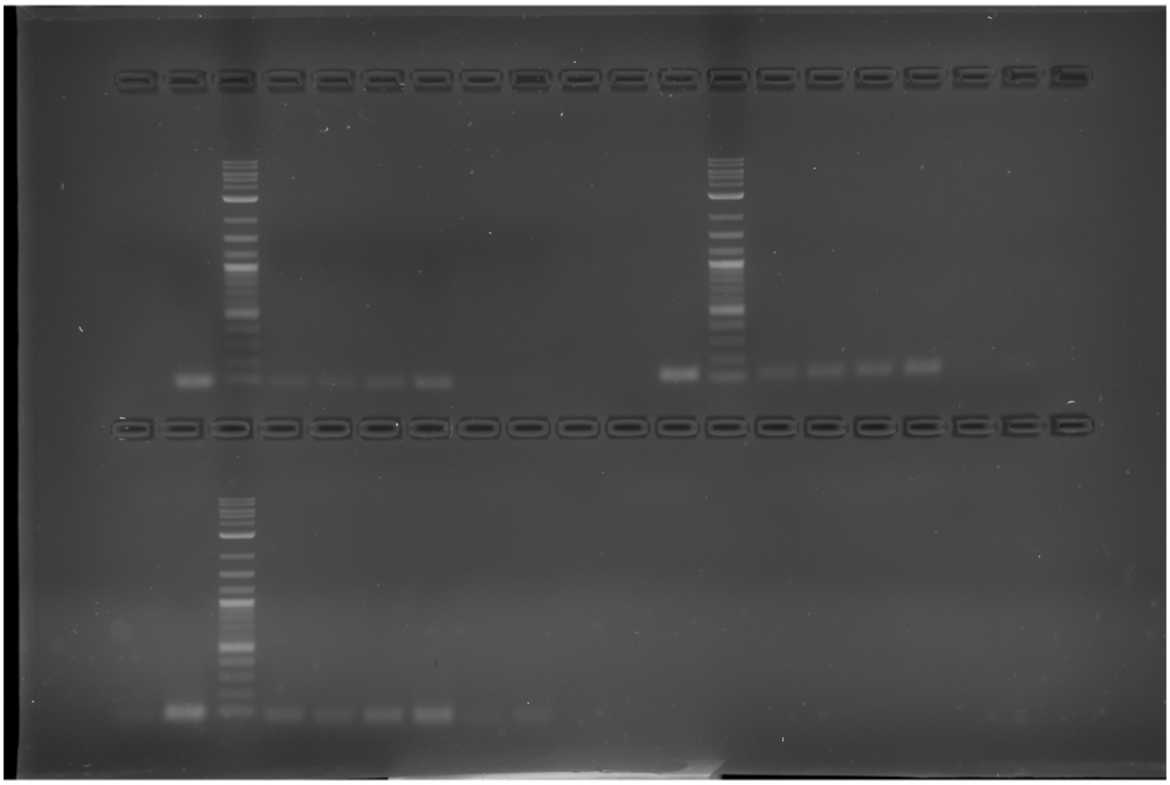

GFP MMP28 GFP MMP28 GFP MMP28

ChIP-1 **ChIP-2** **ChIP-3**

Site3

The gel is stained with XpertGreen according to manufacture protocol. Samples are mixed with 6x loading Orange-G. Imaged in Auto mode in a BioRad GelDoc Imaging System
