# Supplementary material for: Paracrine regulation of neural crest EMT by placodal MMP28
Source: PLoS Biol. 2023 Aug 17;21(8):e3002261. doi: 10.1371/journal.pbio.3002261 (PMC10479893; doi:10.1371/journal.pbio.3002261)
Supplement: Attachment — Submitted filename: 119272_1_rebuttal_2590852_rpt5s6.docx [file pbio.3002261.s020.docx]

**Manuscript number:** RC-2021-00981

**Corresponding author(s):** Eric, Theveneau

## 1. General Statements [optional]

*This section is optional. Insert here any general statements you wish to make about the goal of the study or about the reviews.*

## 2. Point-by-point description of the revisions

*This section is mandatory.* *Please insert a point-by-point reply describing the revisions that were already carried out and included in the transferred manuscript.*

**Dear Editor,**

**We were pleased to see that the reviewers found the study to be “*clearly written*”. The reviewers deemed the work to be a “*pioneer study*” providing “*compelling evidence*” and “*sufficient control experiments*” to support the findings which represent a “*significant advance in the field*” with “*significant implications on the field of EMT*”. They also found that the work would be of “*particular interest to audiences interested in cancer, cell, and developmental biology, as it highlights the importance of the non-canonical function of metalloproteinases during EMT and migration*” and that “*the target audience goes without doubt beyond developmental biologists and includes cell and cancer biologists, and any biologist interested by MMPs or cell invasion mechanisms in vivo*.”**

**We would like to thank the reviewers for taking the time to provide detailed reviews of our manuscript. Please find below our point-by-point answers to their comments. We made our best effort to fully revise the manuscript and we hope you will find it suitable for publication.**

------------------------------------------------------------------------------
**Reviewer #1 (Evidence, reproducibility and clarity (Required)):**

*This manuscript by Gouignard et al., reports that a matrix metalloproteinase MMP28 regulates neural crest EMT and migration by transcriptional control rather than matrix remodeling. The manuscript is clearly written and provides sufficient evidence and control experiments to demonstrate that the MMP28 can translocate into nucleus of non-producing cells and that nuclear localization and catalytic activity are essential for the activity of MMP28 to regulate gene transcription. ChIP-PCR analysis also suggests that MMP28 can bind to the proximal promoters of Twist and others. However, since weak binding is also detected between MMP14 and the promoters, a more direct evidence that such binding can indeed promote Twist expression will be more appreciated.*

**Thank you for this comment. First, to represent the data from our ChIP assays we normalized all intensities to the GFP condition such that all levels are expressed fold change to GFP and we performed statistical comparisons. This shows that the enrichment of promoter regions by MMP28 and MMP14 are not equivalent.**

**Second, to substantiate our previous ChIP data, we performed a new set of ChIP experiments, by performing three independent chromatin immunoprecipitations (biological replicates), and used primers targeting three new domains in the proximal promoter of Twist and primers against two domains in the proximal promoter of E-cadherin and one domain 1kb away from transcription start of E-cadh. We found that pull down with MMP28 significantly enriches the three tested domains within the proximal promoter of Twist but not those of the E-cadherin promoter, compared to GFP pull down. These data were added to Figure 7.**

**However, we do not propose that MMP28 might act as a transcription factor and be able to promote Twist expression on its own. We apologize if some of the initial description of our data were too blunt and might have misled the reviewers. First, the protein sequence of MMP28, like those of all other MMPs, does not contain any typical DNA binding sites. In addition, ectopic overexpression of MMP28 is not sufficient to promote ectopic Twist expression (as shown in supplementary Figure 4) whereas, by contrast, Twist is able to promote ectopic expression of Cadherin-11 (see new Supplementary Figure 11). This indicates that MMP28 has an effect on Twist expression in the context of neural crest only and is not capable of activating Twist expression by itself.**

**Also, it should be added that enrichments of promoter domains by MMP28 pull-down are very modest in comparison to enrichments obtain with Twist pull-downs. Therefore, a more plausible role for MMP28 is to be part of a regulatory cascade with other factors involved in regulating the expression of the target genes important for EMT. Other MMPs such as MMP14 and MMP3 have been shown to interact with chromatin with some transcriptional downstream effects but multiple domains of these proteins seem to equally mediate such interactions. None of the data published in these studies rules out a relay via cofactors. We extensively modified the text describing our data and provided additional context.**

**Identifying the putative partners and their functional relationship with MMP28 is a project on its own and beyond the scope of this study.**

While the nuclear translocation and transcription regulation activity of MMP28 is clearly the focus of the study, there are some minor issues that should be further clarified in the functional studies in the earlier part of the manuscript.

First, the effect of the splicing MO is somewhat unexpected. I would think that the splicing MO would lead to the retention of intron one and therefore premature termination or frameshift of the protein product, but RT-PCR or RT-qPCR suggest that there is no retention of intron 1, but a reduction in the full-length transcript, exon 1, or exon 7-8. Why is that?

**Thank you for this comment. This is presumably due to nonsense mediated RNA decay. We have not explored the biochemistry of MMP28 RNA following injection with MOspl. Splicing MOs can have multiple effects. As explained on the GeneTools website splicing MOs disturb the normal processing of pre-mRNA and cells have various ways to deal with this and there are multiple possible outcomes. The PCR with E1-I1 suggests that intron 1 is not retained. Therefore, a putative concern would be that MOspl led to exon-skipping and to the generation of a truncated form of MMP28. However, we have checked that it is not the case. The fact that the PCR using E7-E8 primers indicates a reduction as well suggests an overall degradation of the mRNA for MMP28. Importantly, the effect of MOspl can be rescued using MMP28 mRNA indicating that the knockdown is specific.**

Second, the effect of the splicing MO and ATG MO in NC explant spreading seems to be somewhat different, with ATG MO strongly repressed explant spreading, cell protrusion, and cell dispersion, while splicing MO does not affect cell dispersion, but affects the formation of cell protrusions. Does this reflects different severity of the phenotype or does the product of splicing MO display some activity?

**Thank you for this comment. However, we think that there may be a confusion. Data on Fig2 (MOatg) and Fig3 (MOspl) both show a decrease of neural crest migration *in vivo* (Figure 2a-b) and of neural crest dispersion *ex vivo* (Fig2c, Fig3i-k). Along the course of the project we have never observed a difference in penetrance or intensity of the phenotypes between the two MOs.**

Also, the switch between ATG MO and splicing MO is a bit confusing, maybe it is better to keep splicing MO only in the main text and move results involving ATG MO to supplementary studies.

**The reason is purely historical. We had an effect with MOatg that can be rescued but there is no available anti-Xenopus MMP28 to assess its efficiency. So we turned to MOspl to have an internal control of efficiency by PCR. This provides an independent knockdown method reinforcing the findings. Both MOs have been controlled for specificity by rescue with MMP28 and display similar effect on NC migration/dispersion. We see no harm in keeping both in the main figures but if the reviewer feels strongly about this we could perform the suggested redistribution of data between main and supp figures.**

Lastly, in Figure 3C and 3J, it says that the distance of migration or explant areas were normalized to CMO, while normalization against the contralateral uninjected side, or explant area at time 0 makes more sense.

**Thank you for this comment as it will allow us to explain better these quantifications. Regarding *in vivo* measurements (Figure 3c), it is indeed the ratio between injected and non-injected sides that is performed in all conditions and then the ratios are normalized to CMO. We have now clarified this point on all instances throughout the figures.**

**Regarding *ex vivo* measurements (Figure 3j), NC explants are placed onto fibronectin and left to adhere for 1 hour before time-lapse imaging starts. NC cells extracted from MMP28 morphant embryos are not as efficient at adhering and spreading as control NC cells. Therefore, normalizing to t0 would erase that initial difference between control and MMP28 conditions. By normalizing to CMO at t_final we can visualize the initial defect of adhesion and spreading as well as the overall defects since CMO at t_final represents the 100% dispersion possible over the time course of the movie.**

Reviewer #1 (Significance (Required)):

This work provides novel insights of how a metalloprotease that is normally considered to function extracellularly can transfer into the nucleus of neighboring cells and regulate transcription. *This would be of interest to researchers studying EMT, cell migration, and the functions of extracellular proteins in general*. My expertise is in neural crest EMT and migration, and cytoskeletal regulation of cell behavioral changes. I do not have enough background on biochemical analysis.

**Referee Cross-commenting**

I agree with comments from both Reviewers 2 and 3, especially that whether MMP28 regulates placode development (through Six1 expression) should be addressed.

**Reviewer #2 (Evidence, reproducibility and clarity (Required)):**

**Summary:**

In this study, Gouignard et al. beautifully use the Xenopus neural crest as a model system to examine the role of the matrix metalloproteinase MMP28 during EMT. The authors show that mmp28 is expressed by the placodes adjacent to the neural crest. Using in vivo and in vitro perturbation experiments, they show that the catalytic function of MMP28 is necessary for the expression of several neural crest markers, as well as neural crest migration and adhesion. Next, the authors use grafting, confocal imaging, and biochemistry to convincingly demonstrate that MMP28 is translocated into the nucleus of neural crest cells from the adjacent placodes. Finally, nuclear localization of MMP28-GFP is necessary to rescue twist and sox10 expression in MMP28 morphants, and ChIP-PCR experiments suggest direct interactions between MMPs and the proximal promoters of several neural crest genes. These results have significant implications on the field of EMT and highlight an underappreciated role for MMPs as direct regulators of gene expression.

**Major comments:**

Overall, the experiments presented in this study are thoroughly controlled and the results are clearly quantitated and rigorously analyzed. Most claims are well supported by multiple lines of experimental evidence; however, there are a few experiments or observations that this reviewer thinks should be reconsidered for more clarity and accuracy.

1. Supplementary Figure 1 shows the effect of MMP28-MOspl on additional ectodermal markers and shows that there is a significant loss of six1 expression from the placodal domain following MMP28 knockdown. The authors note this as a "slight reduction" on line 95, but since this shows a larger reduction in gene expression than some of the neural crest markers (snai2, sox8, foxd3), this reviewer thinks these results warrant a more significant discussion in this study.

**Thank you for this comment. We apologize for the poor choice of word regarding the description of the effect on Six1 expression. We corrected the associated paragraph.**

**Although we do observe a reduction of Six1 expression upon MMP28 knockdown, this cannot explain the observed downregulation of some neural crest genes in our MMP28 experiments. There are noticeable differences between the effects of Six1 loss of function that have been reported in the literature and the MMP28 knockdown phenotypes we describe. As suggested by the reviewer, we added a paragraph in the discussion.**

Does MMP28 localize to the nucleus of placodal cells as it does with neural crest? If so, is it through interaction with the six1 proximal promoter? If MMP28 does not localize to the nucleus, that would suggest MMP28 function with a different mechanism between epithelial cells distinct from role in EMT. These questions could be addressed by analysis of the placode cells in the images in Figure 5 and use of primers against the six1 proximal promoter on any remaining samples from the ChIP experiment.

**Thank you for this comment. To address whether nuclear entry is specific to the neural crest-placodes interaction, we performed new grafts:**

**- 1/ we replaced neural crest cells from embryos expressing MMP28-GFP by placodal cells injected with Rhodamine-dextran. This generates grafted embryos with control placodes next to placodes overexpressing MMP28-GFP. There, we can analyze entry of MMP28-GFP in placodal cells that do not overexpress it. We detected MMP28 in the cytoplasm and in the nucleus of these placodal cells. However, the rate of nuclear entry was lower than in NC cells.**

**- 2/ To assess the importance of the cell type producing MMP28, we grafted NC cells injected with Rhodamine-dextran next to caudal ectoderm expressing MMP28-GFP. MMP28 was detected in cytoplasm and the nucleus of the NC cells but with a lower efficiency than when NC are grafted next to placodes expressing MMP28-GFP.**

**- 3/ We made animal caps sandwiches with animal caps injected with Rhodamine-dextran and animal caps expressing MMP28-GFP. In this case MMP28-GFP is detected in the cytoplasm but fails to reach the nucleus.**

**Collectively, these data indicate that placodes can import MMP28 produced by placodes and that NC can import MMP28 produced by other cells than placodes. However, in both cases the rate of nuclear entry was lower than in the NC-placode situation. Finally, the animal cap sandwiches indicate that entry into the cells does not predict entry into the nucleus. All these data were added to Supp Figure 7. Statistical comparisons of the proportion of cells with cytoplasmic and nuclear MMP28-GFP in all grafts were added to Figure 5.**

**The Six1 promoter analysis suggested is beyond the scope of this study as our focus is primarily on the role of MMP28 in neural crest development.**

2. In Figure 2c, the authors rescue MMP28-MOatg with injection of MMP28wt mRNA. Does the MOatg bind to the exogenous mRNA? If so, this may just reflect titration of the MOatg. If this is the case, this experiment should be repeated with MOspl instead of MOatg.

**Thank you for this comment. MOatg is designed upstream of the ATG and thus the binding site is not included in the expression construct. We added this important technical information in the methods. Of note, we already have the suggested equivalent of Fig2C with the MOspl on figure 3.**

3. Is there a missing data point in Figure 2d corresponding to the upper bounds of the whisker in the 6 hour time point for the MMP28-MOatg dataset?

**Thank you for pointing this out. The top data point was indeed missing from the graph, and we apologize for this oversight. We have now updated the figure with the correct graph.**

4. The authors present ChIP-PCR results in Figure 7 as the major evidence to support the mechanism of nuclear MMP28 in regulating neural crest EMT through physical interaction with target gene promoters. However, the experimental design and presentation in Figure 7 are somewhat unconventional, and as such, difficult to interpret. First, instead of displaying the band brightness across the gel, the authors should normalize their bands to their negative GFP control, thus allowing for interpretation as a "fold enrichment over GFP control". It would be most clear to present these results in the form of a plot similar to Shimizu-Hirota et al., 2012, Figure 6D. Using qPCR instead of gel-based quantitation would further increase reproducibility by removing any bias in image analysis.

**Thank you for this comment. For each band the value of the adjacent local background was subtracted. We have now normalized to GFP to provide graphs showing the fold change to GFP enrichment as requested.**

**However, we would like to point out that we do not propose that MMP28 might act as a transcription factor and be able to promote Twist expression on its own. First, the protein sequence of MMP28 does not contain any typical DNA binding sites, as is the case for any other MMPs. In addition, ectopic overexpression of MMP28 is not sufficient to promote ectopic Twist expression (see sup figure 4) contrary to Twist that can ectopically induce Cadherin-11 for instance (see sup figure 11). Further, enrichments of promoter domains by MMP28 pull downs are very modest in comparison of the enrichments promoted by Twist pull downs.**

**A more plausible role for MMP28 is that it is recruited via an interaction with other factors involved in regulating the expression of the target genes related to EMT. Identifying the partners and their functional relationship with MMP28 is a project on its own, and beyond the scope of this study.**

Second, a proximal promoter sequence represents only ~250 bp upstream from the transcriptional start site. What is the rationale for testing multiple loci up to 3 kb upstream?

**Thank you for pointing this out. The use of the term “proximal” was indeed misleading we have now corrected this part in the text. Regulatory sequences can be located anywhere so we initially had a broader approach to test for interactions. Following on this reviewer’s comment, we removed the data points corresponding to the very distal sites. In addition, we performed three new independent ChIP-PCR assays with primers in the proximal portion of Twist and E-cadherin promoters and found enrichment in ChIP with MMP28-GFP compared to GFP for Twist but not for E-cadherin (whose expression was not affected by MMP28 knockdown). These data were added to Figure 7.**

It is surprising to see that most of these proteins do not show significant enrichment to a particular locus across this ~3 kb territory, while this reviewer would expect to see enrichment close to the TSS that quickly is lost as you move further upstream. Can you explain why MMP28, MMP14, and often Twist, show similar enrichment across this long genomic region?

**Thank you for this comment. Our initial choice of representation did not allow to compare profiles properly. Fold-enrichment to GFP, as suggested by this reviewer, now shows that Twist, MMP28 and MMP14 do not display the same pattern of enrichment across the various loci and that MMP28 pull downs leads to significant enrichments of some of the domains tested in Cad11 and Twist promoters.**

Third, the authors should include additional genomic loci to act as negative controls. For example, E-cadherin was unaffected by MMP28-MOspl, thus there may be no physical interaction between the E-cadherin locus and MMP28. It would be ideal to display results from at least one neural crest-related and one non-neural crest-related gene. Finally, this experiment requires statistical analyses to increase confidence in these interactions.

**Thank you for this comment. We tested binding to E-cadherin promoter for GFP and MMP28-GFP and found no enrichment with MMP28. We also performed statistics as requested. These data were added to Figure 7.**

**Minor comments:**

1. The authors should expand their abstract to more explicitly describe the experiments and results presented within this study.

**Done**

2. In the introduction, line 57 is unclear. "MMP28 is the latest member..." Is this chronologically? Evolutionarily? After this, the authors' statement that the roles of MMP28 are "poorly described" (lines 59-60) seems contradicting with their next sentences citing several studies that document the roles of MMP28 in diverse systems.

**Thank you for this comment. The term “poorly described” was meant with respect to other MMPs with more extensive literature. We have now rephrased this part. Regarding the “latest member” we meant the last to be identified. We have now rephrased this part.**

3. To increase clarity, the authors should define which cell types are labeled by in situ hybridization for sox10 and foxi4.1 in Figure 1e.

**Thank you, we performed the requested clarifications and expanded the change to add the cell types labelled by the other genes used on the figure (see figure legend).**

4. The PCR analysis for mmp28 splicing shown in Figure 1g is very clear and well demonstrates the efficacy of the MMP28-MOspl. However, the authors should note in the figure legend what the "ODC" row represents as this is unclear.

**We added the definition of ODC in the figure legends and in the methods.**

5. On line 118 the authors first reference "MOatg" but should explicitly define this reagent and its mechanism of action for clarity.

**We performed the requested clarification.**

Reviewer #2 (Significance (Required)):

This study by Gouignard et al. provides compelling evidence for the role of MMP28 during neural crest EMT. As neural crest cells share similar EMT and migration mechanisms with cancer progression, they represent a powerful system in which to study these biological processes in vivo. Previous work on MMP function has focused primarily on extracellular matrix remodeling and the effect on cell migration, with less attention given to the role of MMPs during EMT. More recent reports in other systems have begun to elucidate a role for MMP translocation into the nucleus, indicating a surprising and novel mechanism for these proteins. This work would be of particular interest to audiences interested in cancer, cell, and developmental biology, as it highlights the importance of the non-canonical function of metalloproteinases during EMT and migration.

**Referee Cross-commenting**

As with Reviewer #1, I was surprised that the RT-PCR analysis presented in support of the splicing MO lacked retention of intron one. I reasoned this might be due to reduced transcript abundance through a mechanism such as nonsense-mediated decay, but I agree that this data raises questions that the authors should address.

**Thank you for this comment. Indeed, this is presumably due to nonsense mediated RNA decay. We have not explored the biochemistry of MMP28 RNA following injection with MOspl. Splicing MOs can have multiple effects. As explained on the GeneTools website splicing MOs disturb the normal processing of pre-mRNA and cells have various ways to deal with this and there are multiple possible outcomes. The PCR with E1-I1 suggests that intron 1 is not retained. Therefore, a putative concern would be that MOspl led to exon-skipping and to the generation of a truncated form of MMP28. However, we have checked that it is not the case. The fact that the PCR using E7-E8 primers indicates a reduction as well suggest an overall degradation of the mRNA for MMP28. Importantly, the effect of MOspl can be rescued using MMP28 mRNA indicating that the knockdown is specific.**

I also agree with the other comments from Reviewers 1 and 3.


**Reviewer #3 (Evidence, reproducibility and clarity (Required)):**

**Summary**

This study by Gouignard and colleagues explores the mechanisms involving the matrix-metalloprotease MMP28 in the epithelial-to-mesenchymal transition (EMT) of neural crest cells. Interestingly and provocatively, they focus not only on the extracellular functions of this protease but also on the roles of MMP28 in the nucleus. This in non-conventional sub-cellular localization is shared with other MMPs, but its significance remains poorly understood. Here, the authors show that the nuclear function of MMP28 impacts the expression of key EMT regulators in neural crest cells in vivo.

Using Xenopus laevis as a powerful animal model to explore the early development, the authors show that mmp28 expression is found in the ectodermal placodal tissue adjacent to the neural crest prior and after EMT.
In the first part of the study, the authors show that MMP28 depletion affects a subset of neural crest marker gene expression (snai2, twi, sox10) but not others (sox9, snai1), suggesting a specific role on a subset of the genes important for neural crest EMT. The MMP28 depletion phenotype is restored by coinjecting MMP28 MO and MMP28 mRNA, provided that the catalytic activity of the encoded protein is maintained. Next, epistasis (rescue) experiments show that Twist1 can compensate MMP28 depletion.
The second part of the study elegantly shows that MMP28 produced by host adjacent tissues can translocate into the nucleus of neural crest cells grafted from a donor embryo (devoid of MMP28-GFP expression). It also shows that MMP28 nuclear localization as well as its catalytic activity are both required for activating the neural crest gene twist1 and sox10; and that MMP28 is found bound on the chromatin of twist1, cad11 and sox10.
Altogether, these experiments strongly support a model for the nuclear role of MMP28 in the activation (or maintenance) of key genes of the EMT program in vertebrate neural crest cells.

**Major comments**

The key conclusions are:

Conclusion 1: MMP28, expressed and secreted by placodes, is important for complete neural crest patterning prior to EMT, including activation of twist1 and EMT effector cadherin 11 genes. MMP28 is important for neural crest EMT and migration in vivo and in explant assay in vitro.

However, this conclusion omits potential indirect effect of interfering with placode formation itself, as indicated by the strong decrease in six1 expression in morphant embryos. The effect of MMP28MO on the expression of six1 is as strong as for neural crest markers snai2, twi, for example. Line 95, "slight reduction" should be modified.

**Thank you or this comment. We have now modified the associated text.**

What this may mean for placodal development itself, as well as for indirect effects on neural crest cells need to be discussed.

**Following this comment, we added a paragraph in the discussion about Six1.**

Conclusion 2: Gain of Twist 1 (but not Cadherin 11) rescues MMP28 morphant phenotype, allowing EMT to occur and restoring several parameters of cell migration in vivo and in explant assay

Conclusion 3: When secreted from adjacent cells, MMP28 is translocated into the nucleus of neural crest cells and displays a nuclear function important for the activation of twist1 expression.

Both conclusions 2 and 3 are supported by multiple elegant and convincing experimental data. These conclusions do not depend on mmp28 exclusive expression by the placodal ectoderm, and would still be important if there was a minor expression in the neural crest cells themselves (and thus an autocrine effect).

Additional experiments to strengthen the conclusions
Related to Conclusion 1:

- line 102-106: In the rescue experiment, is six1 expression rescued too?

**Thank you for this comment. As detailed in the newly added discussion paragraph about the effects of Six1 loss of function that have been described in the literature, it is very unlikely that our NC phenotypes stem from the observed reduction of Six1 expression.**

**Nonetheless, following this comment we checked for Six1 expression in the placodal domain following MMP28 knockdown and rescue condition. In the rescue condition, only 25% of the embryos had recovered Six1 expression in placodes while 75% of the embryos recovered Sox10 expression in neural crest cells. These data further confirm that rescue of placodal genes is not a pre-requisite for the rescue of neural crest genes and were added in Supp Figure 5.**

**Although MMP28 is likely to have a role in placodes as well, the expansion of Sox2 and Pax3 expression domain and the loss of Eya1 expression typically associated with Six1 knockdown did not occur in MMP28 knockdown. Our story being focused on neural crest cells, we did not investigate further how the MMP28-dependent effect on Six1 might impair placode development.**

- Figure 2g: qPCR analysis suggests that mmp28 is expressed in the neural crest explants themselves, levels being lowered by the MO injection. The levels of this potential expression in the neural crest itself should be compared to the levels in the placodal ectoderm. How do the authors exclude an effect of the MO within the neural crest tissue, independently of roles from the placodal tissue?

**Thank you for this comment. There is a very small subpopulation of NC cells called the medial crest that expresses MMP28. They are along a thin line along the edge of the neural folds. We previously described this in Gouignard et al Phil Trans Royal Soc B 2020. It is useful for us as an internal control for MO efficiency but the expression in placodes is much stronger and involves many more cells. However, this expression called our attention at the onset of the project and we performed some experiments to assess whether some of the observed effects were due to a NC-autonomous effect, as suggested by this reviewer. To test for this we performed targeted injected of the MO such that the medial crest would receive the MO but not the placodes. Targeting the medial crest with MMP28-MO had no effect on Sox10 expression. These data were added to new supp Figure 1.**

The cost and time for these additional experiments is limited (about 3 weeks), and uses reagents already available to the authors.

Data and Methods are described with details including all necessary information to replicate the study. Replication is carefully done and statistical analysis seems convincing.

**Minor comments**

Experimental suggestions to further strengthen the conclusions.
Related to Conclusion 1: - Figure 1e, frontal histological sections would help distinguishing between placodal tissue and neural crest mesenchyme.

**Thank you for this comment. We previously published a detailed expression pattern with such sections (Gouignard et al Phil Trans Royal Soc B, 2020). We rephrased the text to better refer to this previous publication.**

Related to Conclusion 2: - Figure 3: in explants co-injected with twist1 mRNA, is cad11 expression restored? Could this indicate if cad11 is (or is not) part of the program controlled by Twist1 (as suggested by the last main figure)?

**Thank you for this comment. We checked for Cadherin-11 expression in control MO, MMP28-MOspl and MOspl+Twist mRNA and Twist is indeed capable of inducing Cadherin-11 and even leads to ectopic activation of Cad11 on the injected side. These data were added to new Supp Figure 11.**

Related to Conclusion 3: is MMP28 translocation seen in any cell context? Could the authors repeat experiments in Figure 6a with animal cap ectoderm? And with sandwich animal cap ectoderm, one expressing MMP28-GFP versions (wt, deltaSPNLS) and the other Rhodamine Dextran only? This would allow to generalize the mechanism or on the contrary to show a neural crest specificity.

**Thank you for this comment. Following this suggestion and comments from the other reviewers, we performed new grafting experiments.**

- **1/ we replaced neural crest cells from embryos expressing MMP28-GFP by placodal cells injected with Rhodamine-dextran. This generates grafted embryos with control placodes next to placodes overexpressing MMP28-GFP. There, we can analyze entry of MMP28-GFP in placodal cells that do not overexpress it. We detected MMP28 in the cytoplasm and in the nucleus of these placodal cells. However, the rate of nuclear entry was lower than in NC cells.**

**- 2/ To assess the importance of the cell type producing MMP28 we grafted NC cells injected with Rhodamine-dextran next to caudal ectoderm expressing MMP28-GFP. MMP28 was detected in cytoplasm and the nucleus of the NC cells but with a lower efficiency than when NC are grafted next to placodes expressing MMP28-GFP.**

**- 3/ We made animal caps sandwiches with animal caps injected with Rhodamine-dextran and animal caps expressing MMP28-GFP. In this case MMP28-GFP is detected in the cytoplasm but fails to reach the nucleus. These data indicate that placodes can import MMP28 produced by placodes and that NC can import MMP28 produced by other cells than placodes. However, in both cases the rate of nuclear entry was lower than in the NC-placode situation. Finally the animal cap sandwiches indicate that entry into the cells does not predict entry into the nucleus. All these data were added to new Supp Figure 7 and quantifications of import of MMP28-GFP in the cytoplasm and the nucleus all conditions added to Figure 5.**

In supplementary figure 4a, the grey (RDx) is not visible in the zoom in images.

**As the grey channel interferes with visualizing the green channel, we only show the grey channel on the first low magnification image so that the position of grafted cells can be seen. We found it better to omit it from the zoomed in images to avoid masking the GFP signal.**

In figure 7a,b MMP14 is green, GFP is grey (mentioned wrongly in line 276)

**Thank you for pointing this out. We have extensively modified Figure 7 and such issues are now resolved.**

Bibliographical references are accurate. Clarity of the text and figures is excellent, except maybe Figure 7, where a qPCR analysis would be easier to visualize, especially with low-level or fuzzy bands on the gel.

**Thank you. We have now modified Figure 7, including normalization to GFP to show fold-change enrichment and have added new data from three independent ChIP assays for proximal Twist and E-cadherin promoters that we hope further substantiate our initial observations.**

Reviewer #3 (Significance (Required)):

**Place of the work in the field's context:**

In cancer, the MMP proteins are widely described in multiple tumor contexts and promote cell invasion. In development, several studies have focused on their functions in the extracellular space. The nuclear localization of MMP family proteins has been described previously but remained poorly understood so far. This work is thus a pioneer study aiming to understand MMP28 nuclear function.

**Advance:**

This study makes a significant advance in the field, by unraveling the importance of the MMP28 activity in the cell nucleus for the expression of key EMT regulators. Moreover, the study suggests that extracellular MMP28 secreted by adjacent cells or tissues can be internalized and transported to cell nucleus into cells located several cell diameters away. This study thus supports a novel facet of MMP proteins activity, complementary to their previously described role on the extracellular matrix, and further favoring cell invasion, in development and potentially in cancer too.

The target audience goes without doubt beyond developmental biologists (the primary interest) and also includes cell and cancer biologists, and any biologist interested by MMPs or cell invasion mechanisms in vivo.

My field of expertise is developmental biology focused on neural and neural crest early development, mainly using animal models in vivo and some cell culture experiments. I also focus on some aspects of cancer cell migration.
