# Supplementary material for: Paracrine regulation of neural crest EMT by placodal MMP28
Source: PLoS Biol. 2023 Aug 17;21(8):e3002261. doi: 10.1371/journal.pbio.3002261 (PMC10479893; doi:10.1371/journal.pbio.3002261)
Supplement: Attachment — Submitted filename: Response to Reviewers.docx [file pbio.3002261.s022.docx]

**Response to reviewers**

*Rev. 1: Shuyi Nie - note that this reviewer has signed her review*

*The authors addressed most of my previous comments and have included additional experiments to clarify their conclusions and improve the manuscript.*

*There is just one minor point regarding the previous comment on Figure 3j. I agree that if the time it takes for explants to attach and flatten varies, then comparing to T0, which is one hour after placing the explants may lead to misleading conclusions. However, comparing the area of explants with the area of control explants at the end of the experiment has to presume all explants are of exactly the same size. However, no matter how careful the explants are dissected, the variations in explant size in inevitable, as shown by the diverse range in Figure 3k. Given this variation, the explants receiving MMP28-MOspl and Cad11 could possibly be about half the size of control explants and spread equally well as control explants as reflected by the graph. My suggestion is to start measuring explant area right after plating, and compare the area of the same explant from its new time 0, so that the dynamic change can be captured*.

**Regarding explants size, we show that the size and position of the neural crest domain are normal in embryos in which MMP28 has been knocked down, as analyzed by snail1 and Sox9 expression (see Figure 1k). In addition, once dissected out, neural crest populations are systematically chopped down in smaller pieces before being transferred in the petri dish for time-lapse imaging. Larger samples are cut in more pieces than smaller ones. This procedure limits any impact of initial variation in size that may occur naturally between embryos. We clarified this in the methods section.**

**Regarding the dynamic of spreading, it is monitored by plotting variations of areas over time which is what we have shown in Figure 3J. There are two aspects that distinguish MMP28-MO and MMP28-MO + cad11 conditions from the controls and rescue with Twist.**

**The first one is the slope of the curve. The slope is what represents the dynamics of spreading and this slope is not influenced by normalization at t0 or t_final. It reflects the changes from one time point to another within each condition. The slope is steeper in CMO and MMP28-MO + twist conditions than for the MMP28-MO and MMP28-MO + cad11 conditions indicating that the dynamic of spreading is impaired in these last two conditions compared to controls and the rescued condition with Twist. As explant spreading is driven by cell motility, we further assessed the dynamics of cells by performing manual tracking of single cells located at the border of explants for each conditions. Tracking data show that motility is impaired in MMP28-MO and MMP28-MO+Cad11 but not in the MMP28-MO + twist condition compared to control MO cells. We added these data on Figure 3 (panel 3l) and added a paragraph about tracking in the methods section.**

**The second one is the stalling of cells in MMP28-MO and MMP28-MO + cad11 conditions. This is substantiated by the representative time-lapse movies provided where the dynamics of cells and explants indicate that MMP28-MO cells and MMP28-MO + cad11 cells fail to sustain motility throughout the footage. Migration eventually comes to a halt sooner in MMP28-MO cells and MMP28-MO + cad11 cells than in the control MO and MMP28-MO + Twist conditions which leads to a stall in spreading represented by the curves for the MMP28-MO and MMP28-MO+cad11 conditions reaching a plateau at around 4.5 hours whereas CMO and MMP28-MO + Twist conditions continue spreading. This is in line with our observations that cells in MMP28-MO and MMP28-MO + cad11 fail to adhere normally to the substrate (Figure 3 e-h)**

**In conclusion, impaired spreading of explants in MMP28-MO and MMP28-MO + cad11 comes from two problem: a lower cell motility than control cells and an inability to sustain motility for as long as the control cells.**

*Rev. 2:*

*This report by Gouignard et al. examines the function of the matrix metalloproteinase MMP28 in regulating epithelial-to-mesenchymal transition (EMT) in the Xenopus neural crest. The authors show that MMP28 is expressed by the placodal cells, is secreted, and subsequently internalized by the adjacent neural crest. Using in vivo and in vitro perturbations, the authors demonstrate that MMP28 catalytic function is necessary for neural crest marker expression, migration, and adhesion. Further experiments convincingly demonstrate that MMP28 from the placodes is translocated into the nucleus of neural crest cells. The authors show that this phenomenon can occur between multiple sending and receiving cell populations but occurs most robustly between the placode-neural crest. Finally, rescue experiments indicate that MMP28 nuclear localization is necessary to rescue neural crest marker expression, and thorough ChIP-PCR experiments suggest direct interactions between MMP28 and neural crest genes.*

*This manuscript is clearly presented, and I commend the authors on the new experiments, analyses, and text revisions that have carefully addressed the points raised by myself and the two other previous reviewers from Review Commons. As such, I find this manuscript to be suitable for publication in PLOS Biology and believe it will significantly impact future EMT studies.*

**We would like to thank all reviewers for their feedback and suggestions that have helped strengthen this study.**
